# Complete Blood Count and Monocyte Distribution Width–Based Machine Learning Algorithms for Sepsis Detection: Multicentric Development and External Validation Study

**DOI:** 10.2196/55492

**Published:** 2025-02-26

**Authors:** Andrea Campagner, Luisa Agnello, Anna Carobene, Andrea Padoan, Fabio Del Ben, Massimo Locatelli, Mario Plebani, Agostino Ognibene, Maria Lorubbio, Elena De Vecchi, Andrea Cortegiani, Elisa Piva, Donatella Poz, Francesco Curcio, Federico Cabitza, Marcello Ciaccio

**Affiliations:** 1 IRCCS Ospedale Galeazzi Sant'Ambrogio Milan Italy; 2 University of Palermo Palermo Italy; 3 IRCCS San Raffaele Scientific Institute Milano Italy; 4 Department of Medicine University of Padova Padova Italy; 5 Laboratory Medicine Unit University-Hospital of Padova Padova Italy; 6 IRCCS Centro Di Riferimento Oncologico Aviano Aviano Italy; 7 San Donato Hospital of Arezzo Arezzo Italy; 8 University Hospital Policlinico Paolo Giaccone Palermo Italy; 9 Azienda Socio Sanitaria Territoriale di Mantova Mantova Italy; 10 University Hospital of Udine Udine Italy; 11 Department of Computer Science, Systems and Communication University of Milano-Bicocca Milano Italy

**Keywords:** sepsis, medical machine learning, external validation, complete blood count, controllable AI, machine learning, artificial intelligence, development study, validation study, organ, organ dysfunction, detection, clinical signs, clinical symptoms, biomarker, diagnostic, machine learning model, sepsis detection, early detection, data distribution

## Abstract

**Background:**

Sepsis is an organ dysfunction caused by a dysregulated host response to infection. Early detection is fundamental to improving the patient outcome. Laboratory medicine can play a crucial role by providing biomarkers whose alteration can be detected before the onset of clinical signs and symptoms. In particular, the relevance of monocyte distribution width (MDW) as a sepsis biomarker has emerged in the previous decade. However, despite encouraging results, MDW has poor sensitivity and positive predictive value when compared to other biomarkers.

**Objective:**

This study aims to investigate the use of machine learning (ML) to overcome the limitations mentioned earlier by combining different parameters and therefore improving sepsis detection. However, making ML models function in clinical practice may be problematic, as their performance may suffer when deployed in contexts other than the research environment. In fact, even widely used commercially available models have been demonstrated to generalize poorly in out-of-distribution scenarios.

**Methods:**

In this multicentric study, we developed ML models whose intended use is the early detection of sepsis on the basis of MDW and complete blood count parameters. In total, data from 6 patient cohorts (encompassing 5344 patients) collected at 5 different Italian hospitals were used to train and externally validate ML models. The models were trained on a patient cohort encompassing patients enrolled at the emergency department, and it was externally validated on 5 different cohorts encompassing patients enrolled at both the emergency department and the intensive care unit. The cohorts were selected to exhibit a variety of data distribution shifts compared to the training set, including label, covariate, and missing data shifts, enabling a conservative validation of the developed models. To improve generalizability and robustness to different types of distribution shifts, the developed ML models combine traditional methodologies with advanced techniques inspired by controllable artificial intelligence (AI), namely cautious classification, which gives the ML models the ability to abstain from making predictions, and explainable AI, which provides health operators with useful information about the models’ functioning.

**Results:**

The developed models achieved good performance on the internal validation (area under the receiver operating characteristic curve between 0.91 and 0.98), as well as consistent generalization performance across the external validation datasets (area under the receiver operating characteristic curve between 0.75 and 0.95), outperforming baseline biomarkers and state-of-the-art ML models for sepsis detection. Controllable AI techniques were further able to improve performance and were used to derive an interpretable set of diagnostic rules.

**Conclusions:**

Our findings demonstrate how controllable AI approaches based on complete blood count and MDW may be used for the early detection of sepsis while also demonstrating how the proposed methodology can be used to develop ML models that are more resistant to different types of data distribution shifts.

## Introduction

Sepsis is a life-threatening organ dysfunction caused by a dysregulated host response to infection [1]. The progression from infection to sepsis is influenced by both host and pathogen features and, accordingly, is characterized by a high interindividual variability. Sepsis represents a medical emergency, with a strong time dependency, especially for septic shock [2,3]. Therefore, early detection is fundamental to improving the patient outcome by promptly starting treatment. However, sepsis, especially in the early stage, may be characterized by nonspecific signs and symptoms, mainly when diagnosis of infection is still uncertain. Thus, its early recognition is challenging, particularly in the emergency department (ED) and outside the intensive care unit (ICU), due to the lack of data needed to apply the Sepsis-3 criteria (ie, assessment of organ dysfunction due to Sequential Organ Failure Assessment Score [SOFA], plus probable or certain infection). Therefore, reliable tools to screen sepsis are strongly sought after. The UK National Screening Committee underlines that the aim of screening is to propose a test to a population who “do not necessarily perceive that they are at risk of, or are already affected by, a disease or its complications” [4]. In the scenario of sepsis screening, laboratory medicine could have a crucial role by providing biomarkers mirroring the pathophysiological mechanisms underpinning sepsis and whose alteration could be detected before the onset of clinical signs and symptoms [5].

To date, hundreds of biomarkers have been investigated, but several limitations have hampered the translation from research to clinical practice, such as cost and the request for additional blood samples [6]. Intensive scientific research is ongoing to discover accurate, affordable, easy-to-measure, and rapidly available biomarkers for guiding clinicians to identify patients at higher risk of sepsis, allowing close monitoring or early treatment.

In clinical practice, C-reactive protein (CRP) and procalcitonin are the most used sepsis biomarkers. However, they are not specific to sepsis, and their levels can increase in several clinical, noninfectious conditions. In addition, they are usually ordered when sepsis is clinically suspected, leading to a diagnostic delay [7] and making them unsuitable as a screening tool. The ideal biomarker for sepsis screening should have the following characteristics: (1) easy to measure, (2) high sensitivity and negative predictive value for sepsis, (3) low turn-around time, and (4) always available to clinicians, especially when sepsis is not (yet) suspected. Complete blood count (CBC) parameters fulfill all of these features [8]. Indeed, CBC is the first-level laboratory test most ordered in all clinical wards, from ED to ICU. It is easy to perform, cheap, and provides important clinical information on the health status. Beyond the classic CBC parameters, the new generation of hemocytometers provides cell population data, which reflect the morphological and functional characteristics of neutrophils, lymphocytes, and monocytes. In the last decade, the role for monocyte distribution width (MDW) as a sepsis biomarker has emerged [9]. MDW reflects the dispersion around the mean monocyte volume. It is automatically calculated by the last generation DxH hematology analyzer (Beckman Coulter, Inc) based on volume, conductivity, and scattering technology and is provided together with the basic CBC parameters. Noteworthy, MDW received approval from the Food and Drug Administration and European Community In-Vitro Diagnostic Medical Device as an early sepsis indicator in adult patients in the ED. MDW has great potential to be used in clinical practice because it is timely and fruitfully measured without further samples, additional costs, or even a different request from clinicians. Thus, it represents a cost-effective tool for early sepsis screening. This is an important feature in the health care of the third millennium. To date, several authors have shown good performance of MDW for early diagnosis of patients with sepsis in different clinical wards, especially the ED and ICU [10-17]. All studies have shown excellent specificity and negative predictive value (NPV) but suboptimal sensitivity and positive predictive value (PPV) [9]. Thus, while individual CBC biomarkers and MDW represent ideal candidates for sepsis screening in general settings, their performance should be improved to be applied for sepsis screening.

Machine learning (ML) techniques offer the promise to potentially offset the limitations mentioned earlier by combining different parameters, beyond the capabilities of rule-based systems and humans, and increasing the performance of single laboratory parameters [18,19]. Indeed, in recent years, ML methods have been increasingly adopted in health care settings [20], including sepsis screening and prediction [21-24]. However, as also noted in the study by Shashikumar et al [25], most existing ML sepsis models have been developed and validated on data from a single hospital only, with no external validation. Thus, their robustness has not been proven, and their performance may degrade when applied in settings that differ from the one in which they are developed, due to either data or missingness shifts [26], limiting their generalizability and adoption in clinical practice. Indeed, widely applied commercially available models (eg, Epic) have been shown to be severely impacted by the aforementioned issues [27-30]. This is especially true for models based on laboratory parameters, wherein state-of-the-art models have not been externally validated [31]. Most notably, no previous work, to the best of our knowledge, combined the use of ML methods with MDW [32].

According to these premises, the aim of this study is to develop and externally validate ML models whose intended use is the early detection and screening of sepsis on the basis of CBC parameters, including MDW and other clinical and laboratory features commonly available in all clinical laboratories. In this multicentric study we used CBC data from an Italian hospital ED (training set) to develop ML models, by adopting both traditional ML models as well as more advanced techniques inspired by controllable artificial intelligence (AI) [33] (ie, cautious classification and explainable AI) that were successively validated by data from multiple settings (encompassing both ED and ICU settings, as well as different forms of data shifts) and different Italian hospitals, to evaluate their robustness and generalization.

## Methods

### Overview

Herein, we briefly summarize the adopted methodology, which is better detailed in the following sections. The reporting follows the Checklist for Assessment of Medical AI checklist [34] and the “Guidelines for Developing and Reporting Machine Learning Predictive Models in Biomedical Research” [35].

The study was conducted as a multicentric, retrospective study aimed at developing ML models for the classification problem of sepsis detection from clinical laboratory parameters. To this aim, ML models were trained on data from 1 patient cohort (Palermo, Emergency Department [PA-ED], coming from Palermo, Italy) and evaluated on 5 different patient cohorts (Palermo, Intensive Care Unit [PA-ICU], Padova, Intensive Care Unit [PD-ICU], Udine, Emergency Department [UD-ED], Arezzo, Emergency Department [AR-ED], and Ospedale Galeazzi Sant’Ambrogio, Intensive Care Unit [OGSA-ICU], coming from 5 different hospitals in Italy, respectively from Palermo, Padova, Udine, Arezzo, and Milano [refer to Dataset Collection, Description, and Processing section]), encompassing 5344 records for as many single patients. We decided to consider 5 different external validation cohorts to comprehensively assess the generalizability of the developed models, as well as their robustness to multiple forms of data variation (refer to the subsequent paragraphs). The 6 patient cohorts were collected at 5 different hospitals: 3 cohorts (PA-ED, UD-ED, and AR-ED) were from the ED setting, while the remaining 3 (PA-ICU, PD-ICU, and OGSA-ICU) were from the ICU setting. The PA-ED, PA-ICU, PD-ICU, UD-ED, and AR-ED datasets encompassed 19 features, including the following: demographic information (age and sex), the MDW, other laboratory test results (ie, the CBC, including the differential, and the CRP), together with a derived feature (the neutrophils-lymphocytes ratio). The OGSA-ICU dataset encompassed the same features as in the other cohorts, except for the MDW feature that was missing. The PA-ED cohort was used for training, hyperparameter optimization, and internal validation, while all the other cohorts were used only for external validation. The 5 external datasets allowed for the validation of the robustness of the developed models under different shift settings, including covariate shifts due to different clinical settings (ICU vs ED), label shifts (sepsis-2 vs sepsis-3), missingness shifts [26], and instantial variation [36]. In particular, the AR-ED cohort was used to evaluate the robustness of the developed models in a conservative scenario of label shift (refer to the Dataset Collection, Description, and Processing section), as the population of this dataset only encompassed patients who exhibited clinical signs for suspected sepsis. By contrast, the OGSA-ICU cohort was used to test the performance and robustness of non-MDW systems (that integrate the ML models described earlier) that can also be applied in settings that lack the MDW parameter; indeed, the laboratory parameters for the OGSA-ICU dataset did not contain the MDW feature as they were collected using a Sysmex (rather than the one by Beckmann Coulter, Inc) analyzer. Thus, this cohort was characterized by missingness shift [26] (ie, differences in the distribution of missing values) and differing analytical variation [36] (refer to the Non-MDW Systems Validation section). Notably, no dataset pooling was performed to ensure that the validation sets were entirely blind and guarantee an unbiased estimate of performance and generalizability; hence, each dataset was considered as an entirely separate cohort.

The PA-ED was split into 2 datasets according to a 75/25 split: the largest 75% set was used for training and hyperparameter optimization in cross-validation, while the remaining 25% set was used for internal validation. The split was stratified to preserve the proportion of sepsis and no sepsis cases in the 2 splits of the dataset. Further details on the models’ design and development can be found in the Methods section. We considered 5 different classes of models, namely logistic regression (LR), support vector machine (SVM), random forest (RF), extreme gradient boosting (XGB), and decision trees (DTs). The performance of the models was evaluated according to different criteria, namely: area under the receiver operating characteristic curve (AUC), sensitivity, specificity, PPV, NPV, and average PPV (A-PPV; as measures of discrimination power), Brier score (as a measure of calibration), and the standardized net benefit (sNB, as a measure of utility). Following the state-of-the-art development of medical decision support models for sepsis diagnosis [25], and as a way to increase generalizability and reduce overconfident predictions according to the tenets of controllable AI [33], we also considered *cautious classifiers* based on the trained models (refer to Cautious Classification Methods section). Such models can *abstain* from labeling instances, with the aim of implementing a trade-off between coverage (labels for which a prediction is available) and performance metrics (in particular, sensitivity and PPV). The cautious classifiers were evaluated in terms of their coverage, as well as with respect to the high-confidence (HC) metrics, which simply consider the metrics defined earlier only on the subset of the nonabstained-upon cases.

### Dataset Collection, Description, and Processing

The PA-ED data refer to patients who were admitted to the ED of the University Hospital “Policlinico Paolo Giaccone” between September, 4, 2019, and November 30, 2019, as described in the study by Agnello [37]. The inclusion criteria were adult (aged ≥18 years) patients presenting to the ED, and whose initial evaluation included a CBC. Exclusion criteria were (1) aged <18 years, (2) incomplete data collection, (3) discharge from the ED within 2 hours from the ED presentation, (4) readmission to the ED within 12 hours, (5) inadequate blood samples (eg, analyzed >4 hours after collection), (6) failure to determine the MDW parameter, and (7) diagnosis of a hematological disorder. The sepsis diagnosis was made according to sepsis-2 criteria [38]. Demographical, clinical, and laboratory data of patients were recorded from the electronic health records of the hospital. Laboratory parameters were measured upon ED admission. MDW and CBC parameters were measured on blood samples collected in the EDTA-K3 tube by a UniCel DxH 900 hematology analyzer (Beckman Coulter, Inc) within 2 hours of collection, as recommended by the manufacturer, after the laboratory analysis ordered by clinicians was performed. CRP and procalcitonin were measured on serum by a fully automated platform (Cobas 8000, Roche Diagnostics).

The PD-ICU data refer to patients who were admitted to the ICU of the University Hospital of Padova and were collected between January 13, 2020 and April 8, 2020. The inclusion criteria were adult (aged >18 years) patients presenting to the ICU, who remained hospitalized for at least 24 hours, enrolled no more than once, with a CBC and differential testing performed at presentation and over the entire course of the length of stay, as part of standard medical care, and procalcitonin or CRP tests ordered at the same time. Exclusion criteria were an incomplete data collection, a failure to determine MDW at least once, and patients with hematological disorders. Sepsis diagnosis was made according to the sepsis-3 criteria, using the SOFA score. Septic shock was recognized in the cases with hypotension requiring vasopressors and with an elevated serum lactate (>2 mmol/L).

The PA-ICU data refer to patients who were admitted to the ICU of the University Hospital “P. Giaccone” between September 10, 2019, and March 19, 2020, as previously described in the study by Agnello et al [8]. The inclusion criteria were all consecutive adult (aged ≥18 years) patients who were admitted to the ICU. Exclusion criteria were (1) individuals aged <18 years; (2) incomplete data collection; (3) failure to determine the MDW parameter; and (4) underlying conditions potentially associated with deregulation of the immune system, including AIDS, organ or bone marrow transplantation, and hematologic diseases. Sepsis diagnosis was made according to sepsis-3 consensus criteria [1]. Laboratory parameters were measured upon ICU admission. MDW and CBC parameters were measured on blood samples collected in the EDTA-K3 tube by a UniCel DxH 900 hematology analyzer (Beckman Coulter, Inc) within 2 hours of collection, as recommended by the manufacturer, after the laboratory analysis ordered by clinicians was performed. CRP and procalcitonin were measured on serum by a fully automated platform (Cobas 8000, Roche Diagnostics).

The AR-ED data refer to patients who were admitted in the ED of the San Donato Hospital and were collected between July 5, 2019, and April 17, 2020. The inclusion criteria were all consecutive adult (aged ≥18 years) patients who were admitted to the ED and had a clinical presentation of suspected sepsis (at least 2 of the following signs: SOFA≥2, body temperature ≥38 °C or ≤36 °C, infection, respiratory rate ≥20 beats/min, heart rate ≥90 beats/min, or altered mental status). Sepsis diagnosis was made according to the sepsis-3 criteria. MDW and CBC parameters were measured by a UniCel DxH 900 hematology analyzer (Beckman Coulter, Inc) within 2 hours of collection and directly extracted from the analyzer. CRP was measured by a Cobas 8000 instrument (Roche Diagnostics) and manually extracted from the hospital’s laboratory information system (LIS).

The OGSA-ICU data refer to patients who were admitted to the ICU of the Istituto di Ricovero e Cura a Carattere Scientifico Ospedale Galeazzi Sant’Ambrogio between January 1, 2023 and April 31, 2023. The inclusion criteria were adult (aged ≥18 years) patients who were hospitalized in the ICU for at least 2 days (to exclude patients who were temporarily admitted to the ICU because of specific intervention protocols) and for whom blood culture and procalcitonin were requested. All hematological parameters were measured by a Sysmex XN2000 blood analyzer and manually extracted from the hospital’s LIS (BeSimpleLIS, Siemens SpA), using QlikView (Qlik, Inc). CRP and procalcitonin were measured by the Atellica IM Analyzer (Siemens) and manually extracted from the hospital’s LIS using QlikView.

The UD-ED data refer to patients who were admitted to the ED of the University Hospital of Udine between November 1, 2019, and December 31, 2019. The inclusion criteria were adult (aged ≥18 years) patients who were enrolled in the ED and had a CBC exam (with differential) requested as part of the standard diagnostic pathway. Sepsis diagnosis was made according to the sepsis-2 criteria. MDW and CBC parameters were measured by a UniCel DxH 900 hematology analyzer (Beckman Coulter, Inc), and directly extracted from the analyzer. CRP was measured by the Cobas C analyzer (Roche Diagnostics) and manually extracted from the hospital’s LIS.

### Model Design and Development

We considered 5 different classes of statistical machine learning models, namely LR, SVM, RF, XGB, and DTs. These model classes were selected as they exhibited state-of-the-art in-sample [39] and generalization [40] performance on diagnostic tasks from laboratory data in previous work; particularly, we decided to not focus on deep learning models due to several limitations of these latter models on tabular data tasks [41-43], such as requiring extensive hyperparameter optimization and having subpar generalization performance.

The ML models were implemented in Python, using the scikit-learn library (version 1.0.2; Python Software Foundation). Particularly, all models were implemented as 3-step pipelines encompassing a feature scaling step (the scaling technique was selected as a hyperparameter during model training), a feature selection step (using the recursive feature elimination method with an LR model for feature scoring), and the classification step. The full set of hyperparameters, for all the evaluated models, is available in Table S1 in Multimedia Appendix 1. Particularly, except for model-specific hyperparameters, the set of common hyperparameters encompassed the scaling method to be used, the number of features to be selected, and class weights to manage label imbalance.

Model training, hyperparameter selection, and internal model validation were performed on the PA-ED dataset. Specifically, model training and hyperparameter selection were performed on the training fold of the dataset (which encompassed 75% of the total number of cases), while internal validation was performed on the test fold of the same dataset (which encompassed 25% of the total number of cases). Hyperparameter selection was performed by means of randomized search, with a budget of 1000 evaluations, in a 5-fold stratified cross-validation design (RandomizedSearchCV). The training set was split into 5 folds (each containing 20% of the cases in the training data and 15% of the total number of cases in the PA-ED dataset). Following this, for each of the 1000 randomized search evaluations, a random set of hyperparameters was selected and then the models with the specified hyperparameter values were evaluated by means of a 5-fold cross-validation procedure by which they were trained on 4 of the cross-validation folds and evaluated on the remaining fold. Stratified cross-validation was used to preserve the proportion of sepsis and no sepsis cases across all folds. Each evaluated hyperparameter configuration was scored by the average AUC score across the 5 cross-validation folds. For each model class, we selected the hyperparameter configuration that had the highest average AUC score.

After hyperparameter selection, the models were retrained on the entire training set and evaluated on the hold-out test set according to different evaluation criteria, namely sensitivity, specificity, PPV, NPV, AUC, A-PPV, Brier score, and sNB (with a threshold equal to 0.5). On the basis of the internal test set, we compared the developed ML models with 4 different baselines: a fixed MDW threshold (threshold value 23.5 [37]), a fixed CRP threshold (threshold value 80 [44]), a fixed Sepsis Index threshold (threshold value 1 [45]), and the state-of-the-art SVM model (MindraySVM) for sepsis diagnosis based on laboratory biomarkers proposed in the study by Aguirre and Urrechaga [31]. In regard to the latter model, due to the different laboratory instrumentation used in our study, we trained an SVM model using the hyperparameter settings specified in the study by Aguirre and Urrechaga [31] on the same training data used for our models. We also compared the developed models with the state-of-the-art Conformal Multidimensional Prediction of Sepsis Risk (COMPOSER) model proposed in the study by Shashikumar et al [25].

### External Validation

The developed ML models were subsequently externally validated, after retraining on the entire PA-ED dataset, on the 4 external validation cohorts (AR-ED, PA-ICU, PD-ICU, and UD-ED), in terms of the same evaluation criteria used for internal validation. As described earlier, the considered external validation allowed us to evaluate the generalizability of the developed ML models in a variety of settings that differed in terms of hospital (4 different hospitals across different regions in Italy), reference population (ED vs ICU), as well as diagnostic criteria (sepsis-2 vs sepsis-3). Therefore, we decided to keep the external validation datasets separate from the internal training set (PA-ED) and did not perform any data pooling. In particular, the choice to consider 4 external validation datasets (as well as an additional external dataset for validation in settings with missing MDW) enabled us to comprehensively assess the robustness of the developed models under a varied selection of distribution shifts. We also evaluated the similarity between the PA-ED cohort and the 5 external validation cohorts, using the degree of correspondence metric (ψ) proposed in the study by Cabitza et al [46]. The ψ metric is a similarity measure used to compare 2 datasets and provides a *P* value for the hypothesis test that 2 datasets come from the same distribution. The ψ metric has been used as a way to evaluate the robustness of ML models in the context of out-of-domain generalization [40] by assessing the ability of ML models to report good performance also on datasets with low ψ value. We used the implementation of the ψ metric provided [47]. In the paper we used the ψ metric to assess the similarity between the training set (PA-ED) and the external validation cohorts, as well as how the performance of the models varied with respect to the value of ψ.

### Validation of Non-MDW Systems

Because the MDW feature was not available in the OGSA-ICU cohort (missingness rate: 100%), we developed non-MDW ML systems for use in settings that lack the MDW parameter. These systems encompassed the ML models trained on the PA-ED cohort, without any retraining, as well as a preprocessing imputation model used to fill in the missing MDW values. The imputation model was derived from the data from the other cohorts (PA-ED, AR-ED, PA-ICU, PD-ICU, and UD-ED), which were used as a training set. The MDW was considered the target feature, while all the other features were used as predictors. The imputation model was an RF regression model with default hyperparameters and was implemented in Python using the scikit-learn library (version 1.0.2). To evaluate the goodness of fit of the imputation model, the training set was split into folds according to a 75/25 split: the 75% was used to fit the imputation model, while the remaining 25% was used for model evaluation. The performance of the imputation model was assessed by means of the adjusted *R*^2^ score computed between the predicted and true MDW values. After assessment, the imputation model was retrained on the entire training dataset and applied as a preprocessing step to the OGSA-ICU dataset to impute the MDW values for subsequent analysis.

The non-MDW systems were then evaluated by adopting the same methodology described in the Model Design and Development section on the OGSA-ICU cohort. Specifically, we simply applied the developed ML models to the imputed data from the OGSA-ICU cohort.

### Cautious Classification Methods

To enable the models to detect a control loss [33] and abstain from providing a diagnosis, to control error rates and reduce misdiagnosis, we implemented cautious classification models based on the ML models trained on the PA-ED cohort. Cautious classification refers to a set of uncertainty quantification techniques that enable an ML model to abstain from providing a prediction, for a given case, whenever the model is not sufficiently confident [48]. Cautious classification has been successfully applied for error rate control in recent work [49,50], also in the context of sepsis diagnosis [25], and provides a simple approach to improve the performance and perceived utility of an ML model. Specifically, we adopted a simple, thresholding-based strategy to implement the cautious classification models: given any new case, the cautious classifiers provided the same diagnosis as the corresponding ML model if the confidence provided by this latter was >0.75; otherwise, it *abstained* from providing a diagnosis. Thus, for a given case, if the ML model would associate with the sepsis class a confidence score larger or equal to 0.75, the cautious classifier would also classify that case as sepsis; similarly, if the confidence score for sepsis was <0.25 (ie, the confidence score for the absence of sepsis was >0.75), the cautious classifier would classify the case as no sepsis; finally, if the confidence score was between 0.25 and 0.75, then the cautious classifier would be considered as abstaining from providing a diagnosis. We decided to apply this cautious classification strategy, rather than other approaches, such as 3-way decision [51] or conformal prediction [52], due to its increased efficiency (the computational complexity cost of the thresholding strategy is O(1), while it is on the order of O(log n), for n being the dataset size, for conformal prediction), ease of interpretation, and its equivalence, in the binary classification setting and under weak assumptions, with the 2 aforementioned methods [51]. Regarding the implementation of the cautious classifiers, we used the HC metrics implemented in the scikit-cautious library [53], and applied them to the ML models trained on the PA-ED dataset, setting the threshold hyperparameter (th) to 0.75. The HC metrics evaluate the performance of the models on the set of nonabstained cases; hence, they select the cases for which either the confidence score of sepsis or that of no sepsis is >0.75. We considered, particularly, the HC metrics for PPV, NPV, sensitivity, specificity, AUC, A-PPV, Brier score, and sNB, as well as the coverage (the percentage of nonabstained-upon cases).

### Statistical Analysis

For all continuous variables, we reported mean and SD, while for all categorical variables, we reported the frequency of each value. The sNB was computed at the fixed threshold value of 0.5. CIs for sensitivity, specificity, PPV, and NPV were calculated using the Wald CI formula [54], namely,



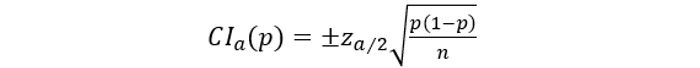

**(1)**


where *p* is the estimated value, *n* is the sample size, and *z*_a/2_ is the *(1- a/2)* quantile for the standard normal distribution. CIs for the AUC, A-PPV, and sNB were calculated based on the formulas defined in the study by Riley et al [55] for estimation of the SE and Wald CI formula, namely,



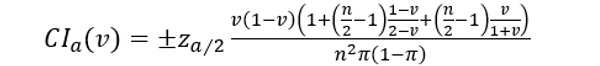

**(2)**




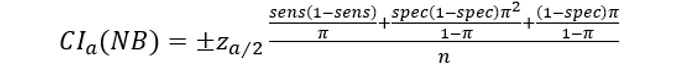

**(3)**


where *v* is the estimated AUC or A-PPV value, *π* is the proportion of patients with sepsis in a given cohort, and *sens* (respectively, *spec*) is the estimated sensitivity (resp. specificity). CIs for the Brier score were computed based on Hoeffding inequality [56] for bounding the difference between the sample estimate of a continuous, bounded random variable and its expected value, namely:



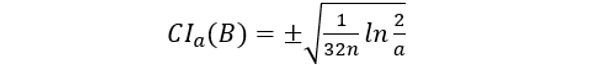

**(4)**


For each pair of developed ML models and each evaluation metric, we evaluated the hypothesis that the 2 classifiers had the same (null hypothesis) or different average performance: we used a 2-tailed chi-square test to test the hypotheses and computed the *P* values. For each developed ML model and each dataset, we evaluated the hypothesis that the cautious classifier provided an improvement in terms of sensitivity, specificity, PPV, or NPV compared to the corresponding standard ML model: we used a 1-tailed chi-square test to test the hypotheses and compute the *P* value. Finally, for each developed ML model, each external validation dataset, and each evaluation metric, we evaluated the hypothesis that the performance of the ML model was not worse than what was reported on the internal validation dataset. We used a 2-tailed chi-square test to test the hypotheses and compute the *P* values. To control the increased type I error (mistaken rejection of a null hypothesis) due to multiple hypothesis testing, we adjusted the computed *P* values using a false discovery rate correction procedure. Particularly, we used the optimal adaptive procedure by Gavrilov et al [57], which computes the *k-*th adjusted *P* value *p_k_* (with the *P* values sorted in increasing order) as given in the following:



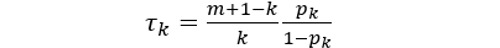

**(5)**




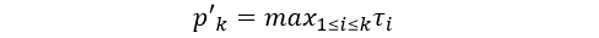

**(6)**


where *α=0.05* is the confidence threshold for null-hypothesis rejection, and *1≤k≤m*, where m was the number of tested hypotheses. All code for statistical analysis was implemented in Python. We used *pandas* and *numpy* for data loading and management, *scipy* for implementation of the chi-square test, and *statsmodels* for implementation of the false discovery rate correction procedure. We used custom Python implementations of the CIs for the AUC, A-PPV, sNB, and Brier score.

### Ethical Considerations

The collection of data at the different sites, as well as the multicentric study, was conducted in compliance with the guidelines established by the local ethics committees, relevant ethical and legal regulations, and in agreement with the World Medical Association Declaration of Helsinki. Regarding the ethics review, the study used only secondary data that had been previously collected at different sites in earlier studies, in accordance with an approved ethical framework from the local ethical boards. The present study does not introduce any new risks, burdens, or interactions with participants beyond those already considered and approved in the original ethical reviews. Since the study did not require any blood draws or procedures from the enrolled patients that would not already have been performed as part of standard medical care, and because the data was rendered anonymous before analysis, written informed consent and approval were exempted by the local committees, and no compensation was provided to the enrolled patients.

## Results

### Overview

The full information about the features’ distribution, as well as the sample sizes, in the 6 datasets is reported in Table 1.

**Table 1:** Characteristics of the populations in the 6 cohorts.

Feature		PA-ED^a^	AR-ED^b^	PA-ICU^c^	PD-ICU^d^	UD-ED^e^	OGSA-ICU^f^
Age (y), mean (SD)		55.1 (20)	69 (21.4)	66.5 (13)	66.3 (14.9)	66.5 (19.2)	64.3 (18.1)
WBC^g^ (10^9^/L), mean (SD)		10.2 (4.1)	11.3 (6.4)	13.9 (6.6)	11.5 (5.4)	8.9 (4.6)	13.4 (6.2)
Neutrophils(10^9^/L), mean (SD)		7.2 (3.9)	8.7 (5.4)	12.7 (6.7)	9.4 (5)	6.4 (3.8)	12.3 (9.4)
Monocytes(10^9^/L), mean (SD)		0.8 (0.3)	0.8 (0.4)	0.8 (0.4)	0.9 (0.5)	0.7 (0.4)	0.8 (0.5)
MDW^h^, mean (SD)		20.1 (3.2)	25.6 (6.4)	24.6 (7.5)	24.1 (5)	21.2 (4.2)	—^i^
CRP^j^ (mg/L), mean (SD)		22.6 (49.3)	83 (93)	81.4 (96.5)	93.9 (81)	25.3 (52.9)	56.6 (80.7)
Lymphocytes (10^9^/L), mean (SD)		2.1 (1.1)	1.6 (3.6)	1 (0.5)	1 (0.6)	1.7 (2.3)	1.2 (1.9)
Eosinophils (10^9^/L), mean (SD)		0.1 (0.2)	0.1 (0.2)	0.1 (0.5)	0.2 (0.4)	0.1 (0.1)	0.1 (0.1)
Basophils(10^9^/L), mean (SD)		0.1 (0.1)	0 (0.1)	0 (0)	0 (0.1)	0 (0)	0.1 (0.1)
RBC^k^ (10^12^/L), mean (SD)		4.6 (0.7)	4.2 (0.8)	3.9 (0.8)	3.5 (0.6)	4.4 (0.7)	3.9 (0.6)
Hemoglobin level (G/100 mL), mean (SD)		13.4 (2.1)	12.3 (2.3)	10.9 (2.3)	10.4 (1.5)	13.1 (2.1)	11.5 (1.8)
Hematocrit test (%), mean (SD)		39.3 (5.9)	37.6 (6.5)	32.6 (7)	31.7 (4.8)	38.8 (5.8)	34.3 (5.1)
MCV^l^ (fL), mean (SD)		86.5 (7.9)	91 (6.7)	85.3 (9.5)	90.1 (6.1)	88.9 (6.4)	89.1 (5.3)
MCH^m^ (pg), mean (SD)		29.5 (3.2)	29.6 (2.4)	29.3 (7.7)	29.5 (2.2)	30.1 (2.6)	29.9 (2.3)
MCHC^n^ (G/100 mL), mean (SD)		34 (1.2)	32.5 (1.1)	33.1 (1.5)	32.7 (0.9)	33.8 (1)	66.1 (327.5)
RDW^o^ (%), mean (SD)		14.6 (2.1)	15.8 (2.4)	48 (7.6)	15.7 (2.9)	14.7 (2.1)	14.5 (2.2)
Platelet count (PLT) (10^9^/L), mean (SD)		254.4 (90.4)	229.6 (105.2)	243.3 (120.8)	236.7 (145.3)	241.7 (88.5)	196.3 (89.5)
NLR^p^, mean (SD)		5 (5.7)	10.3 (11.3)	18 (19.1)	14.4 (17.2)	5.8 (7.1)	16.7 (15.8)
Sex, n (%)							
	Male	870 (48.59)	169 (54.9)	46 (61)	1612 (76.25)	441 (47)	50 (50)
	Female	930 (51.41)	139 (45.1)	29 (39)	502 (23.75)	497 (53)	50 (50)
Sepsis, n (%)							
	Yes	83 (4.59)	76 (24.7)	22 (29)	709 (33.54)	63 (6.7)	6 (6)
	No	1726 (95.41)	232 (75.3)	53 (71)	1405 (66.46)	875 (93.3)	94 (94)
Patients, n (%)		1809 (33.85)	308 (5.76)	75 (1.40)	2114 (39.56)	938 (17.55)	100 (1.87)

^a^PA-ED: Palermo, Emergency Department.

^b^AR-ED: Arezzo, Emergency Department.

^c^PA-ICU: Palermo, Intensive Care Unit.

^d^PD-ICU: Padova, Intensive Care Unit.

^e^UD-ED: Udine, Emergency Department.

^f^OGSA-ICU: Ospedale Galeazzi Sant’Ambrogio, Intensive Care Unit.

^g^WBC: white blood cell.

^h^MDW: monocyte distribution width.

^i^Not available.

^j^CRP: C-reactive protein.

^k^RBC: red blood cell.

^l^MCV: mean corpuscular volume.

^m^MCH: mean corpuscular hemoglobin.

^n^MCHC: mean corpuscular hemoglobin concentration.

^o^RDW: RBC distribution width.

^p^NLR: neutrophils-lymphocytes ratio.

### Internal Validation Results

**Figure 1:**
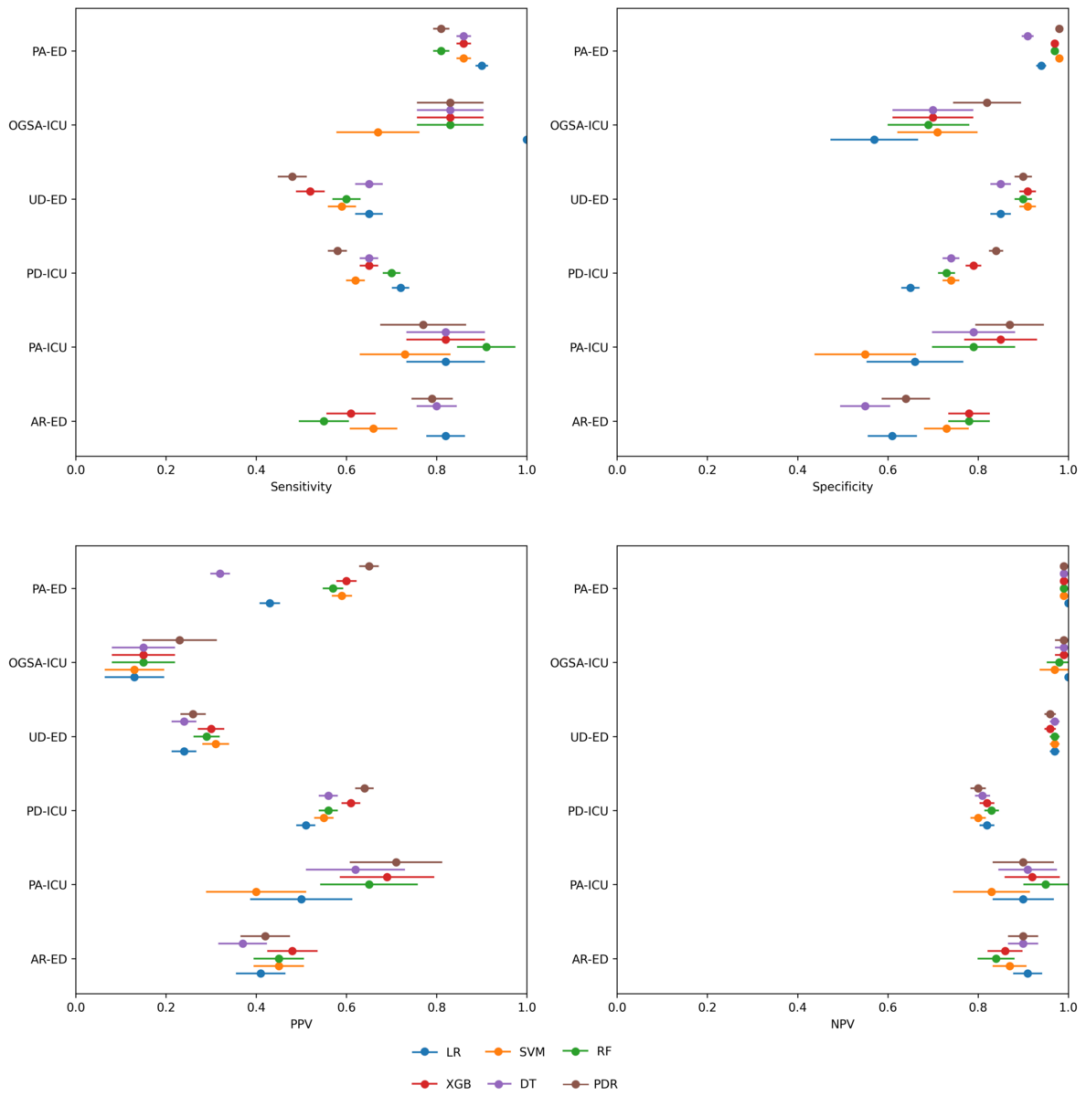
Performance (in terms of sensitivity, specificity, positive predictive value [PPV], and negative predictive value [NPV]) of the developed models, each represented by a colored circle, on the 6 considered datasets (represented on the y-axis), together with the corresponding 95% CIs. AR-ED: Arezzo, Emergency Department; DT: decision tree; LR: logistic regression; OGSA-ICU: Ospedale Galeazzi Sant’Ambrogio, Intensive Care Unit; PA-ED: Palermo, Emergency Department; PA-ICU: Palermo, Intensive Care Unit; PDR: Partial Decision Rule; PD-ICU: Padova, Intensive Care Unit; RF: random forest; SVM: support vector machine; UD-ED: Udine, Emergency Department; XGB: extreme gradient boosting.

**Figure 2:**
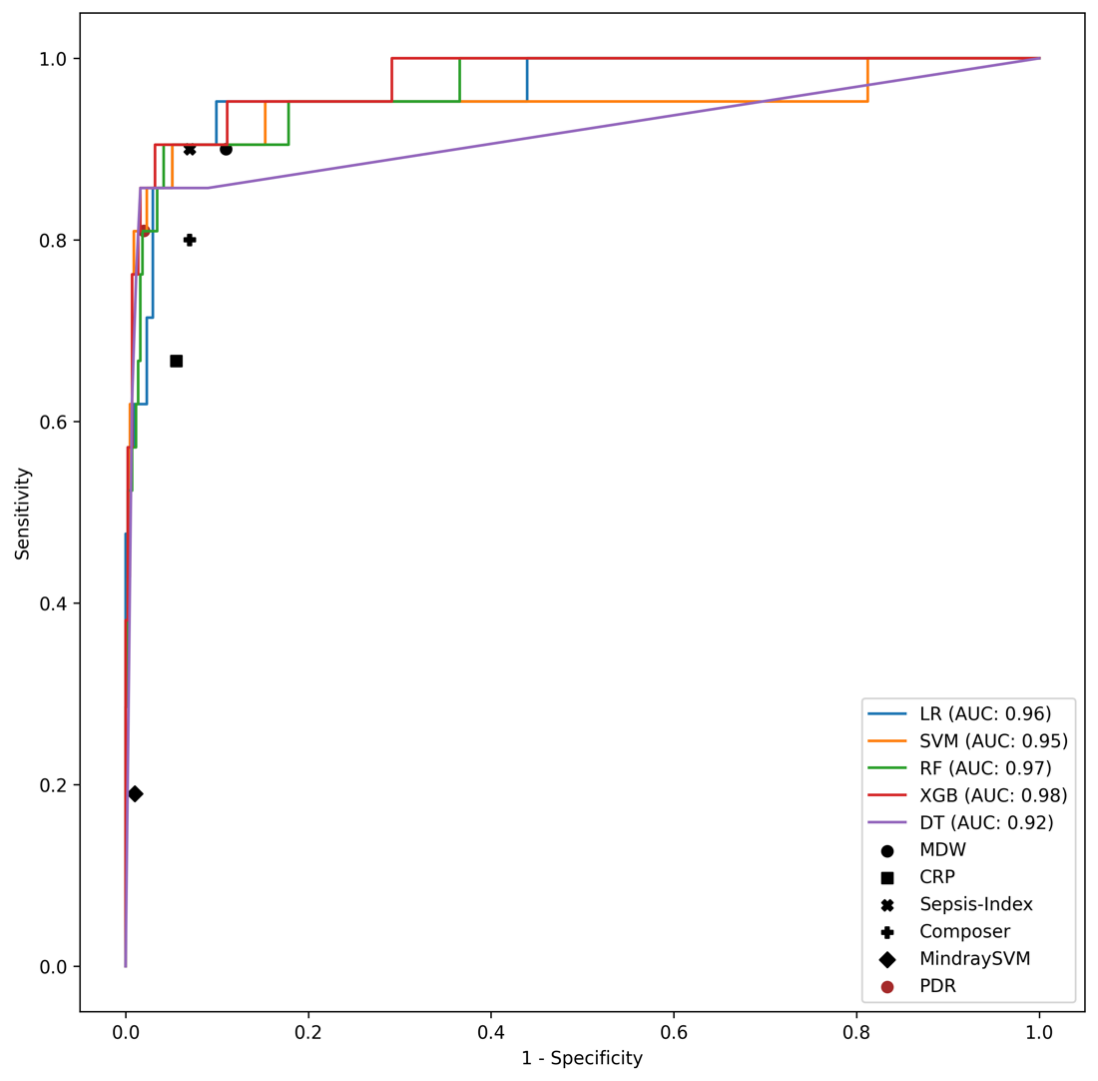
Receiver operating characteristic curves for the models, along with their area under the receiver operating characteristic curve (AUC) values, on the Palermo, Emergency Department (PA-ED) internal validation dataset. In the plot we also report on the sensitivity and specificity of 4 baselines: binary thresholds based on the monocyte distribution width (MDW) [35] and C-reactive protein (CRP) [42], a binary threshold based on the Sepsis-Index parameter [43], the Conformal Multidimensional Prediction of Sepsis Risk model [25], and the support vector machine (SVM) model developed in the study by Aguirre and Urrechaga [31]. DT: decision tree; LR: logistic regression; PDR: Partial Decision Rule; RF: random forest; XGB: extreme gradient boosting.

**Figure 3:**
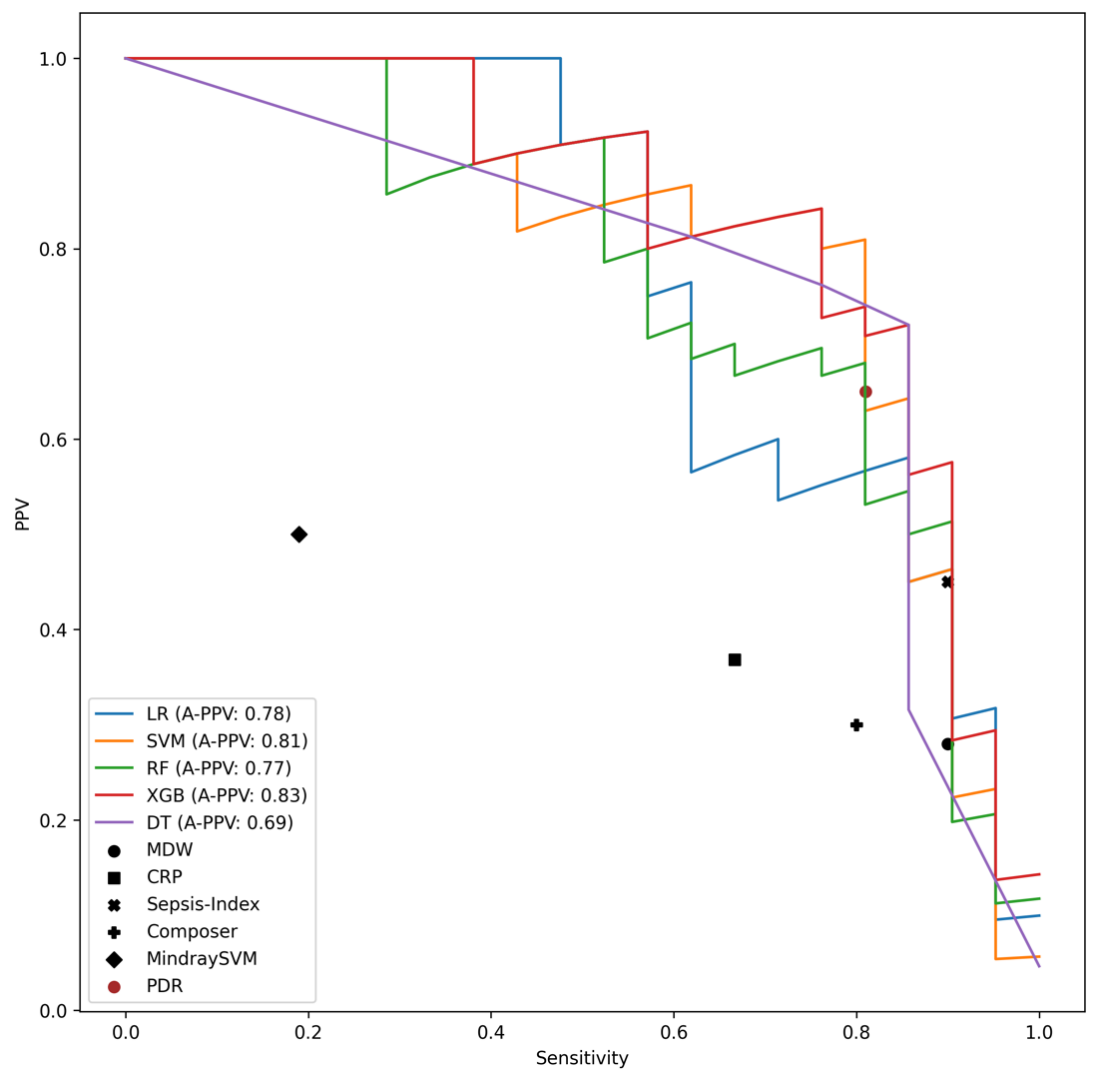
Sensitivity–positive predictive value (PPV) curves for the models, along with their average PPV (A-PPV) values, on the Palermo, Emergency Department (PA-ED) internal validation dataset. The plot also shows the sensitivity and PPV of 4 baselines: binary thresholds based on the monocyte distribution width (MDW) [35] and C-reactive protein (CRP) [42], a binary threshold based on the Sepsis-Index parameter [43], the Conformal Multidimensional Prediction of Sepsis Risk model [25], and the support vector machine (SVM) model developed in the study by Aguirre and Urrechaga [31]. DT: decision tree; LR: logistic regression; PDR: Partial Decision Rule; RF: random forest; XGB: extreme gradient boosting.

**Figure 4:**
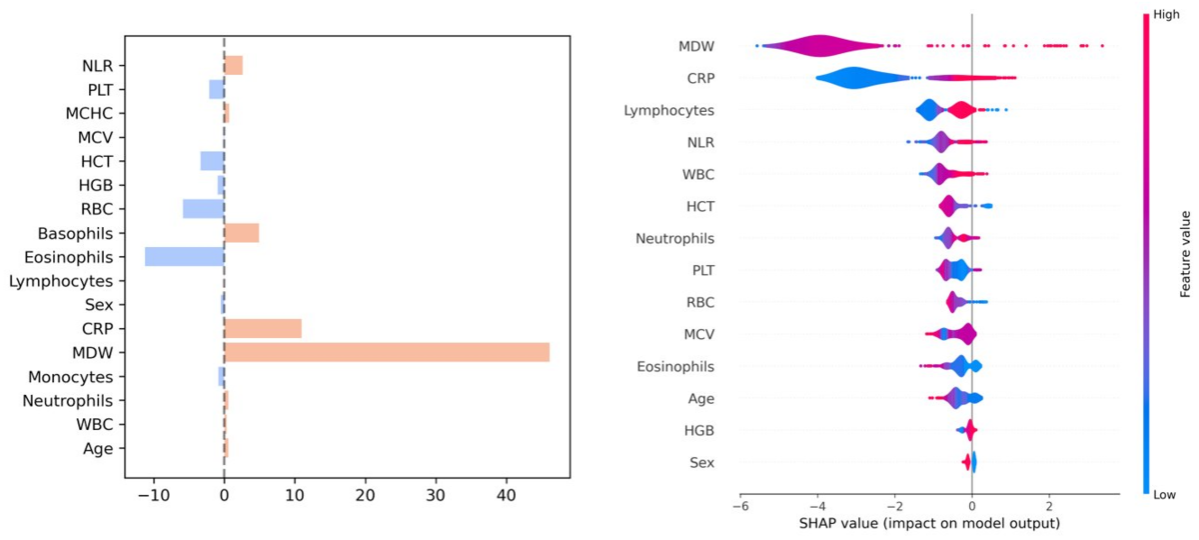
Feature importances for the logistic regression (LR; left) and extreme gradient boosting (XGB; right) models. The feature importance for the LR model represents the coefficients of the induced linear model, shown as bar plots. For each feature, the color represents the sign of the coefficient. The width of the bar represents the magnitude of the coefficient: larger width denotes greater importance. The feature importance for the XGB model was computed through the Shapley Additive Explanations method and is represented in terms of violin plots: for each feature, red denotes high values, while blue denotes low values; values at the right of the middle vertical bar denote an increased confidence score for the positive class (sepsis), while values at the left denote a decreases confidence score. CRP: C-reactive protein; MCHC: mean corpuscular hemoglobin concentration; MCV: mean corpuscular volume; MDW: monocyte distribution width; NLR: neutrophils-lymphocytes ratio; RBC: red blood cell; WBC: white blood cell; HCT: hematocrit test; HGB: hemoglobin; PLT: platelet count.

**Figure 5:**
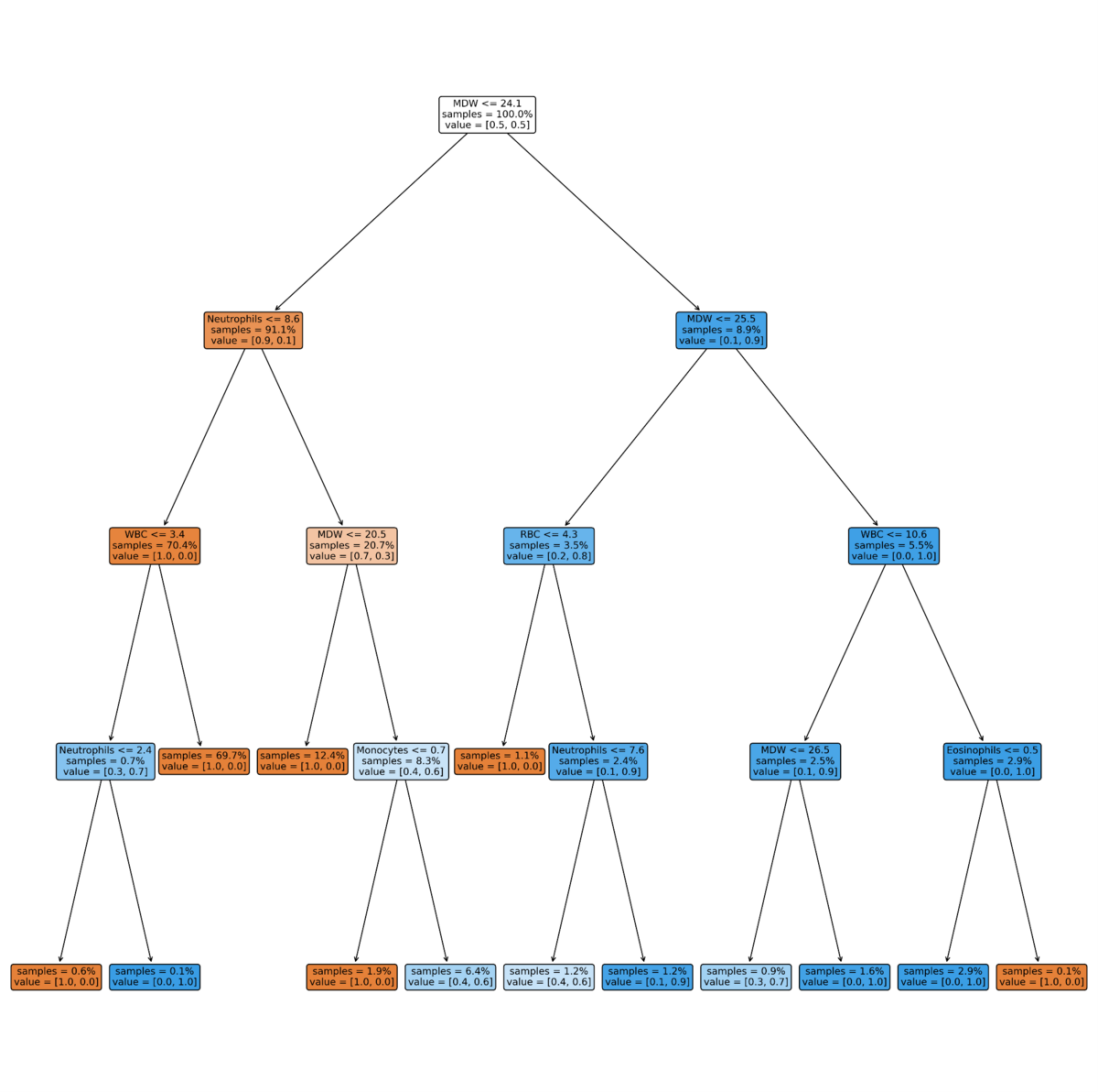
Interpretable decision tree: the color of each node denotes the majority class for the corresponding subset of instances (blue: sepsis and orange: no sepsis). Each non–leaf node contains 3 data elements: a selected feature and corresponding threshold (top); the proportion of samples corresponding to that node (middle); and the proportion of samples belonging to each class (bottom). Leaf nodes contain 2 data elements: the proportion of samples corresponding to that node (top) and the proportion of samples belonging to each class (bottom). MDW: monocyte distribution width; RBC: red blood cell; WBC: white blood cell.

The results of the models on the interval validation set are shown in Table 2 and Figures 1 to 3 (Figure S1 in Multimedia Appendix 1). XGB significantly outperformed all other models in terms of AUC, A-PPV, and sNB (Figure S2 in Multimedia Appendix 1). LR and XGB had greater sensitivity and PPV, respectively, than all other models, although not significantly so. XGB, RF, and LR significantly outperformed all the baseline comparison methods (ie, MDW [37], CRP [44], Sepsis Index [45], COMPOSER [25], as well as the SVM model developed in the study by Aguirre and Urrechaga [31]; Figures 1 and 2; Figure S1 in Multimedia Appendix 1) and did so across all possible decision thresholds. The importance of the most relevant features for the models with the highest AUC and PPV (XGB) and sensitivity (LR) are depicted in Figures 4 and 5. It was identified that MDW was the single most predictive feature (for all models), followed by the CRP, the neutrophils-lymphocytes ratio, the leucocytic formula composition, the hematocrit, and red blood cell count, which also had significant predictive power. An interpretable DT model is given in (Figure 5): after discussion with the clinical experts, we derived from the DT a highly interpretable, cautious decision rule model (Partial Decision Rule [PDR]) defined by the following rules: if MDW>24.1, then sepsis; if MDW ≤24.1 and neutrophils ≤8.6 then no sepsis; if MDW ≤20.5 and neutrophils >8.6 then no sepsis; otherwise abstain.

PDR had better PPV and specificity than the developed ML models, although not significantly so. In terms of sensitivity and NPV, it was significantly worse than only LR. Furthermore, among the nonidentified sepsis cases, most of them (14% of the total number of sepsis cases in the internal test set) were classified by the PDR model classified in the abstain group, while only 1 sepsis case was classified in the no sepsis group.

**Table 2:** The results of the developed machine learning models (with the optimal configuration of hyperparameters) on the internal hold-out test set, together with the corresponding 95% CIs (in parenthesis). The considered models were logistic regression (LR), support vector machine (SVM), random forest (RF), extreme gradient boosting (XGB), decision tree (DT), and the cautious decision rule model (PDR). We also report the performance of state-of-the-art baselines monocyte distribution width (MDW), CRP, Sepsis Index, Mindray SVM, and Conformal Multidimensional Prediction of Sepsis Risk (COMPOSER).

	LR (95% CI)	SVM (95% CI)	RF (95% CI)	DT (95% CI)	XGB (95% CI)	PDR (95% CI)	Mindray SVM (95% CI)	MDW (95% CI)	CRP (95% CI)	Sepsis index (95% CI)	COMPOSER (95% CI)
Sensitivity	0.90 (0.03)^a^	0.86 (0.03)^b^	0.81 (0.04)	0.86 (0.03)^b^	0.86 (0.03)^b^	0.81 (0.04)	0.19 (0.04)	0.90 (0.03)^a^	0.67 (0.04)	0.90 (0.03)^a^	0.80 (0.04)
Specificity	0.94 (0.02)	0.96 (0.01)	0.97 (0.02)^b^	0.91 (0.03)^b^	0.97 (0.02)^b^	0.98 (0.01)^a^	0.99 (0.01)	0.89 (0.03)	0.94 (0.02)	0.93 (0.02)	0.93 (0.02)
PPV^c^	0.43 (0.05)	0.59 (0.04)^b^	0.57 (0.05)^b^	0.32 (0.04)	0.60 (0.05)^b^	0.65 (0.04)^a^	0.50 (0.05)	0.28 (0.04)	0.37 (0.04)	0.45 (0.05)	0.30 (0.04)
NPV^d^	1.00 (0.01)^a^	0.99 (0.01)^b^	0.99 (0.01)^b^	0.99 (0.01)^b^	0.99 (0.01)^b^	0.99 (0.01)^b^	0.96 (0.02)	1 (0.01)^a^	0.98 (0.01)	1 (0.01)^a^	0.98 (0.01)
AUC^e^	0.96 (0)	0.95 (0)	0.97 (0)	0.91 (0)	0.98 (0)^a^	—^f^	0.91 (0)	—	—	—	—
Brier	0.05 (0.02)	0.02 (0.01)^a^	0.03 (0.01)^b^	0.05 (0.02)	0.03 (0.01)^b^	—	0.04 (0.02)^b^	—	—	—	—
A-PPV^g^	0.78 (0.01)	0.81 (0)	0.77 (0.01)	0.67 (0.01)	0.83 (0)^a^	—	0.44 (0.01)	—	—	—	—
HC^h^ sensitivity	0.90 (0.03)^b^	0.81 (0.04)	0.86 (0.03)	0.85 (0.03)	0.90 (0.03)^b^	0.94 (0.02)^a^	0.11 (0.03)	—	—	—	—
HC specificity	0.96 (0.02)	0.98 (0.01)^b^	0.99 (0.01)^a^	0.99 (0.01)^a^	0.98 (0.01)^b^	0.98 (0.01)^b^	0.99 (0.01)^a^	—	—	—	—
HC PPV	0.51 (0.05)	0.62 (0.04)	0.71 (0.04)^b^	0.67 (0.04)^b^	0.72 (0.04)^a^	0.65 (0.04)^b^	0.33 (0.04)	—	—	—	—
HC NPV	0.99 (0.01)^b^	0.99 (0.01)^b^	0.99 (0.01)^b^	0.99 (0.01)^b^	1 (0.01)^a^	1 (0.01)^a^	0.96 (0.02)	—	—	—	—
Coverage	0.94 (0.02)	0.98 (0.01)^b^	0.92 (0.03)	0.92 (0.03)	0.97 (0.02)^b^	0.90 (0.03)	0.99 (0.01)^a^	—	—	—	—
sNB^i^	0.22 (0.01)	0.60 (0.01)^b^	0.35 (0.01)	0.24 (0.01)	0.62 (0.01)^a^	—	0.16 (0)	—	—	—	—

^a^Best performing model.

^b^Models whose 95% CIs overlap with the best model.

^c^PPV: positive predictive value.

^d^NPV: negative predictive value.

^e^AUC: area under the receiver operating characteristic curve.

^f^Not available.

^g^A-PPV: average positive predictive value.

^h^HC: high confidence.

^i^sNB: standardized net benefit.

### External Validation Results

**Figure 6:**
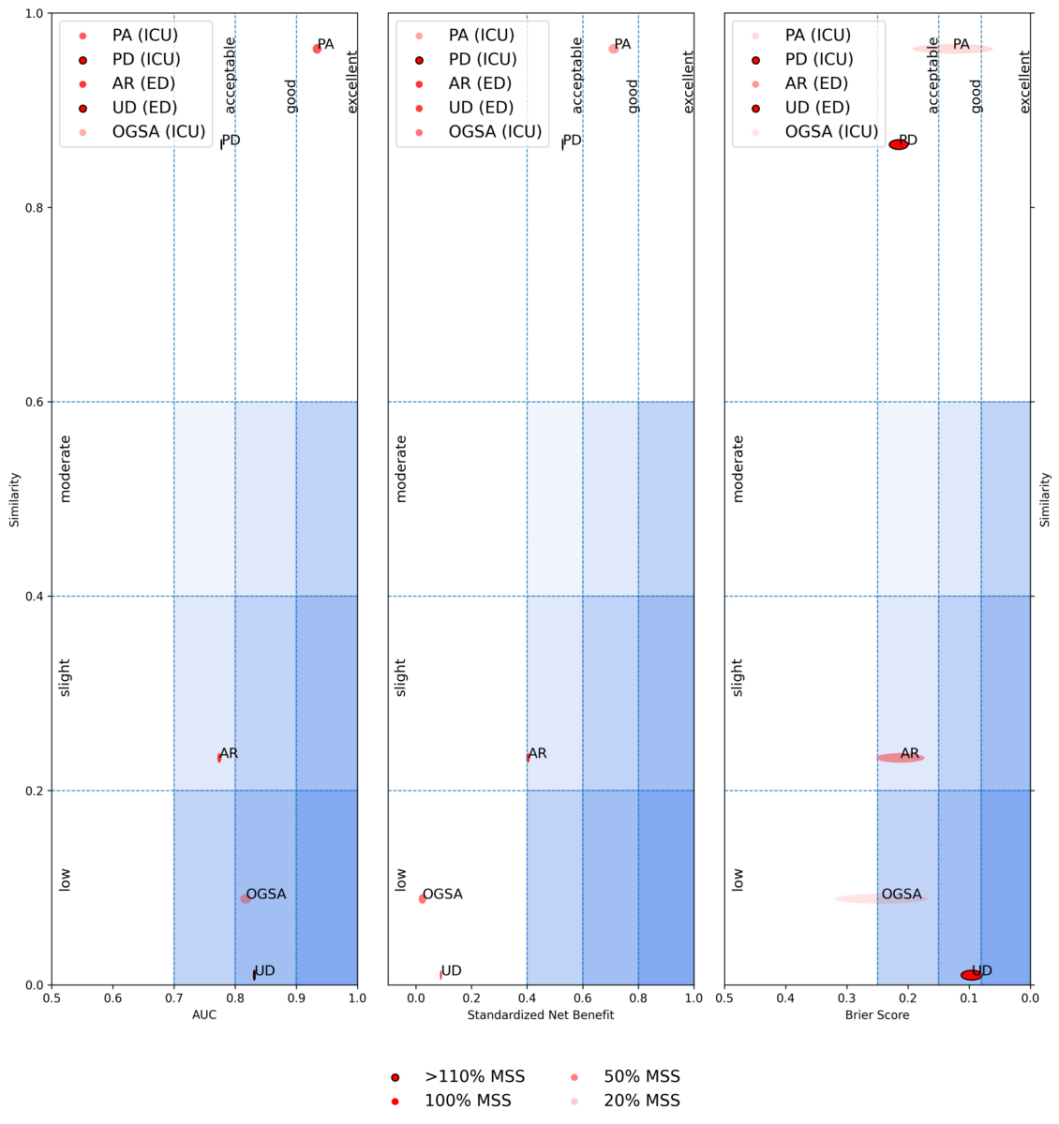
External performance diagram [40] for the extreme gradient boosting (XGB) model on the external datasets. The diagram illustrates the performance of the XGB model according to 3 different aspects: discrimination power (in terms of area under the receiver operating characteristic curve [AUC]), utility (in terms of standardized net benefit), and calibration (in terms of Brier score). The size of the ellipses associated with the datasets denotes the 95% CIs; the transparency of the ellipses denotes the achievement of the minimum sample size (the lower the transparency, the closer the sample size to the minimum sample size). The diagram has been produced with the tool available on the Metimeter website [58]. AR-ED: Arezzo, Emergency Department; OGSA-ICU: Ospedale Galeazzi Sant’Ambrogio, Intensive Care Unit; PA-ICU: Palermo, Intensive Care Unit; PD-ICU: Padova, Intensive Care Unit; UD-ED: Udine, Emergency Department.

The results of all models, including the PDR model, on the external validation datasets (PA-ICU, PD-ICU, UD-ED, and AR-ED) are reported in Table S2 in Multimedia Appendix 1 and Figure 1, as well as in Figures S3 to S6 in Multimedia Appendix 1, in terms of receiver operating characteristic and sensitivity-PPV curves. The results of the external validation for the model with the highest AUC on the internal validation (XGB), are represented in Figure 6 in the form of an external performance diagram [40]. The XGB model achieved acceptable-to-excellent performance on all external datasets and with respect to all metrics, except for the sNB on the UD-ED: this dataset was, among the external validation datasets, the most dissimilar to the internal training set (PA-ED). The external performance diagrams for the other models are in Figures S7 to S10 in Multimedia Appendix 1. While the performance of all models worsened compared with the internal test sets, the differences were not significant, as shown in Figure S11 in Multimedia Appendix 1.

### Non-MDW Validation Results

The data from all cohorts except OGSA-ICU were used to develop an imputation model to predict the value of the MDW feature from all the other considered parameters. The developed imputation model was then used to impute the values of MDW in the OGSA-ICU dataset. The results of the imputation process are in Figure S12 in Multimedia Appendix 1, while additional detail about the implementation of the imputation model is in the Methods section.

The results of the non-MDW systems, on the OGSA-ICU cohort, are reported in Figure 1 as well as in Figure S13 in Multimedia Appendix 1, in terms of ROC and sensitivity-PPV curves. The results of the validation, for the system encompassing the model with the highest AUC on the internal validation (XGB), are represented in Figure 6 in the form of an external performance diagram [40]. The XGB-based system achieved low PPV and sNB on the OGSA-ICU dataset, as also shown in Figure S13 in Multimedia Appendix 1, which was also the second most dissimilar to the internal training set (PA-ED).

### Analysis of Cautious Classifiers

**Figure 7:**
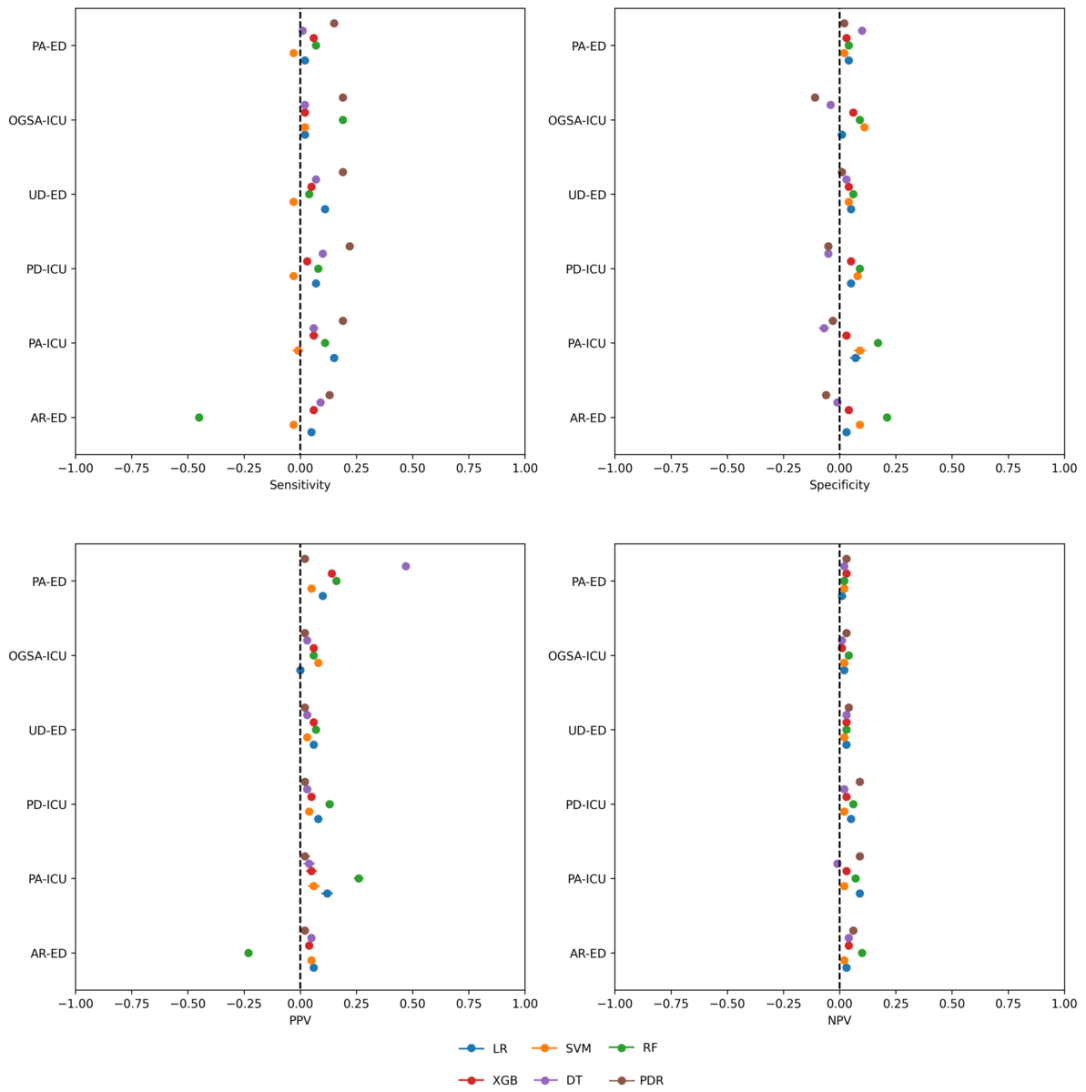
Difference between the performance of cautious models and the corresponding standard models: values greater than 0 denote an improvement in performance in the cautious inference model as compared with the corresponding standard one. The 95% CIs were computed using the pooled SD. Each model is represented by a colored circle. AR-ED: Arezzo, Emergency Department, DT: decision tree; LR: logistic regression; OGSA-ICU: Ospedale Galeazzi Sant’Ambrogio, Intensive Care Unit, PA-ED: Palermo, Emergency Department; PA-ICU: Palermo, Intensive Care Unit; PDR: Partial Decision Rule; PD-ICU: Padova, Intensive Care Unit; RF: random forest; SVM: support vector machine; UD-ED: Udine, Emergency Department; XGB: extreme gradient boosting.

The use of cautious classifiers reduced the amounts of error for almost all models, datasets, and metrics, as is shown in Figure 7 and Figure S14 in Multimedia Appendix 1. For XGB, PDR, RF, and LR (except for the OGSA-ICU dataset), cautious classification led to an improvement in both sensitivity and PPV and equivalent or better performance in terms of NPV. For all models and datasets, the coverage of the cautious classifiers was >50%, and, in all but 3 cases (all for the RF model), >70%. These results indicate that cautious classification could be useful to reduce false negatives, with minimal deleterious effects in terms of the increase of false positives or the number of undecided cases.

## Discussion

### Principal Findings

Sepsis recognition, especially in the ED, is challenging due to the potentially limited data that can be timely collected and difficult logistic settings. The recognition of sepsis triggers the application of validated bundles (ie, fluid resuscitation, antibiotic administration, blood culture and collection of other relevant specimens, and lactate dosage) that can reduce mortality if applied timely [2]. However, there is a risk of overtreatment in the case of *false positive* cases, with potential detrimental effects of therapies and waste of resources.

In this study, we developed and validated 5 ML models, as well as an interpretable set of rules derived from the latter, for sepsis screening based on clinical and laboratory features. The main findings of our study can be summarized as follows: (1) XGB and LR models showed the best performance for sepsis screening; (2) the most important features for the XGB and LR model were MDW and CRP; (3) the ML models have been validated on 4 external datasets, showing good generalizability in terms of both discrimination power, calibration, and utility; (4) the interpretable, cautious decision rules had performance comparable to that of black-box ML models, while being more easily understandable by clinicians; (5) cautious classification techniques could further improve screening performance, with minimal impact on coverage; and (6) while the non-MDW ML systems achieved acceptable performance on the non-MDW validation test, the lack of MDW led to a large decrease in PPV. Noteworthy, 5 datasets were obtained from different Italian hospitals, in different periods, and different clinical wards (namely, ED and ICU), and exhibited widely varying characteristics in terms of covariate, label, and missingness distributions. In particular, the datasets exhibited a large variation in terms of sepsis prevalence in the corresponding populations, also due to differences between ED and ICU settings (wherein the latter have typically larger incidence than the former) or population characteristics (as an example, the OGSA-ICU cohort was obtained from a hospital specialized in orthopedics, a setting that typically has much lower sepsis’ incidence than general hospitals [59]).

### Comparison to Prior Work

In this study, we also compared the developed models against different state-of-the-art approaches, including commonly used clinical thresholds [37,44] and scores [45], as well as ML models [31,32]. Our models reported better performance than all the previous state-of-the-art approaches. In this sense, we believe that the most relevant innovations from the methodological point of view, enabling these results, were the inclusion of MDW in the panel of parameters, which resulted in being the most predictive characteristic for sepsis, and the use of controllable AI methodologies, which enabled us to obtain models that were more robust to various forms of distribution shifts. In this regard, to date, the lack of external validation is one of the most important criticisms of ML studies [40,60]. Indeed, although it is a fundamental step to strengthen model derivation and ensure generalizability, most published articles do not perform it, with potentially negative consequences in terms of robustness, generalizability, and real-world applicability. This issue has been markedly noted also in the context of sepsis screening and prediction, where one of the most commonly adopted proprietary decision support models for sepsis prediction (ie, EPIC) has been shown to be severely impacted by shifts and variabilities associated with varying settings [28-31]. Thus, along with the methodological novelties of our study, one of the main strengths of our study is the external validation on 5 different datasets. On the basis of the results of the external validation, we can conclude that the developed models are able to generalize better than the state-of-the-art, at least in the Italian settings, as they maintain the performance independently from covariate shifts (due to either different patient populations or locations, ED or ICU settings, or different measuring instruments), label shifts (sepsis2 or sepsis3 diagnostic criteria and different incidence of sepsis) as well as missingness shifts scenarios: this latter characteristic, specifically, is of particular interest since, despite its potential impact on model generalization, it has scarcely been investigated in medical ML studies [26]. In this study, we developed ML-based systems that could also be applied in settings where MDW was not available, with the aim of increasing the applicability of the developed ML models as well as of assessing the importance of the MDW parameter (which was the most relevant feature according to all the developed ML models). While, indeed, the obtained results show that acceptable results could be obtained even in the presence of significant missingness shift, the lack of MDW led to a relevant drop in performance, thus highlighting the need for collecting complete data in practical scenarios. By contrast, our models were shown to robustly generalize to different incidence rates, not only due to different clinical settings (ED vs ICU) but also to diagnostic criteria (sepsis-2 vs sepsis-3), allowing us to prove not only their effectiveness and robustness but also that varying incidence rates may have lower influence on performance compared to other variation sources (eg, lack of MDW, as described earlier, or selection criteria, as in the AR-ED cohort).

### Strengths and Limitations

Strengths of this study are the external validation, the large sample size, and the use of several ML methods as well as several indicators to assess the model’s performance. This study comes from the collaboration and cooperation among different professionals, including clinicians, engineers, biologists, and informatics. Each has made its expertise available with the common aim to develop a tool that could be usable in clinical practice.

Limitations of the study must also be mentioned. First, the use of sepsis-2 criteria for sepsis identification in the database used for derivation. However, the use of sepsis-3 criteria in the ED is costly in terms of resources and time-consuming and may be difficult to apply outside the ICU because full assessment of organ dysfunction is needed. Furthermore, the developed models have been shown to generalize well also in settings where sepsis-3 criteria were used for sepsis identification, showing good performance despite this label shift factor. Thus, despite this potential limitation, we believe that the aid of CBC laboratory biomarkers, easily obtainable and at a low cost, may help improve the specificity of both clinical criteria and initial screening and support differential diagnosis in case of negative results. Second, we were not able to stratify patients according to the infectious focus (eg, lung, abdomen, central nervous system, soft tissue, blood, and urinary tract) or clinical severity (sepsis vs septic shock) due to the absence of serum lactate levels in our datasets. Both the type of infection and clinical severity may be potential modifiers of model performance and should be investigated in future work. More generally, lactate levels are important information for sepsis, especially in ICU settings. At the same time, lactates generally lack several characteristics of an ideal biomarker for sepsis screening, being more apt as a marker of severity in patients with high pretest probability of sepsis. The objective of the developed ML models was to provide clinicians with quick but accurate sepsis screening tests, especially in cases where sepsis is not yet suspected (patients accessing the ED): because lactates are not frequently requested during initial management, requiring them as features for our models would severely decrease their use (as an example, none of the 3 ED cohorts considered in our study encompassed lactates). Nonetheless, we believe that future studies should consider the combination of lactate levels and MDW (as well as other CBC parameters), which could potentially lead to even better performance. In addition, it should be highlighted that MDW can be obtained only using a particular hematological analyzer, while other available instrumentations do not allow for the calculation of this interesting index. This aspect is particularly relevant in light of the results of the non-MDW validation test, which highlighted the diagnostic power of the MDW parameter. Indeed, we noted that its lack was associated with a relevant drop in performance (especially as regards the PPV), thus showing the potential advantage to use the mentioned hematological analyzer to collect CBC biomarkers (including MDW). Finally, in this study we only validated our models on retrospective data; further research should evaluate the potential application of a clinical algorithm incorporating the results of our models in prospective studies and for clinical timely decision in screening patients for sepsis.

### Conclusions and Future Directions

In clinical practice, because of their characteristics in performance and robustness, the ML models we developed and presented in this study may represent a reliable tool for supporting physicians in sepsis screening on patients of both sexes admitted to the ED and ICU. Parameters included in the models can be determined rapidly and cost-effectively. Furthermore, the excellent NPV of the models allows us to rule out sepsis with high accuracy, while they are also characterized by a good sensitivity and specificity. Noteworthy, such models significantly improved the specificity and PPV of MDW and CRP alone, as well as of the other considered state-of-the-art ML models, thus confirming our hypothesis that integrating several CBC parameters through the use of ML could significantly improve the performance of traditional biomarkers. Therefore, we conjecture that the developed models could be especially useful as a first-level test to detect sepsis. Accordingly, an increased value (of the confidence score produced by the model) may represent an alert for clinicians that they should perform further investigations to confirm the sepsis suspicion. Indeed, in the case of nonobvious infectious foci, an increased value may help physicians decide to evaluate further biomarkers, such as procalcitonin [61], both fundamental for the appropriate management of patients with sepsis, specifically for diagnosis and guiding treatment, respectively. To this latter aim, the ability to further isolate a group of predictions with higher PPV and good sensitivity, by using the cautious classification models and the interpretable decision rules, is especially interesting. Indeed, from the clinical point of view, it would enable the administration of aggressive therapies in a small sample of patients, but associated with extremely high confidence (90%), thus minimizing the risk of overtreatment and potentially leading to a significant improvement in the outcome. In our opinion, this is a valuable hypothesis to be validated in properly designed prospective trials. At the same time, the cases in which these models abstain could be useful to alert the clinicians of a possible control loss [33] and, hence, reduce the risk of automation bias or de-skilling [62]. In this sense, we conjecture that the developed interpretable decision rules could be particularly fruitful as a rapid and easy-to-use tool to support clinicians in promptly delivering appropriate treatment to the selected patients. Indeed, a recent line of research in the ML literature has noted how the black-box nature of modern ML models, and the consequent adoption of explainable AI techniques (such as model surrogates or feature attribution methods), could potentially increase automation bias (or also other cognitive biases related to the use of automation technology) [63], hence advocating for the use of simple and interpretable models in high-stake domains [64] where humans adopt AI tools in a human-AI teaming scenario [65]. In this sense, the developed decision rule model could address this need while still providing satisfying screening performance. Furthermore, from the clinical point of view, the decision rule model seems to confirm the important role of MDW and neutrophils in sepsis development [66] and provides a first combined screening model based on these 2 parameters that should be further investigated and validated in future studies.

## Appendix

Multimedia Appendix 1 Additional figures and tables reporting the results of the developed machine learning models on both the training and external validation cohorts.

## Abbreviations

AI: artificial intelligence
A-PPV: average positive predictive value
AR-ED: Arezzo, Emergency Department
AUC: area under the receiver operating characteristic curve
CBC: complete blood count
COMPOSER: Conformal Multidimensional Prediction of Sepsis Risk
CRP: C-reactive protein
DT: decision tree
ED: emergency department
HC: high-confidence
HCT: hematocrit test
HGB: hemoglobin level
ICU: intensive care unit
LIS: laboratory information system
LR: logistic regression
MDW: monocyte distribution width
ML: machine learning
NPV: negative predictive value
OGSA-ICU: Ospedale Galeazzi Sant’Ambrogio, Intensive Care Unit
PA-ICU: Palermo, Intensive Care Unit
PD-ICU: Padova, Intensive Care Unit
PDR: Partial Decision Rule
PLT: platelet count
PPV: positive predictive value
RF: random forest
sNB: standardized net benefit
SOFA: Sequential Organ Failure Assessment
SVM: support vector machine
UD-ED: Udine, Emergency Department
XGB: extreme gradient boosting

## Data Availability

The datasets generated during and analyzed during this study are available from the corresponding author on reasonable request. Access to the computer code used in this research is available upon reasonable request to the authors. Code for validation (internal and external), as well as the associated machine learning models, is accessible on the internet [67].
