# Supplementary material for: Complete Blood Count and Monocyte Distribution Width–Based Machine Learning Algorithms for Sepsis Detection: Multicentric Development and External Validation Study
Source: J Med Internet Res. 2025 Feb 26;27:e55492. doi: 10.2196/55492 (PMC11904381; doi:10.2196/55492)
Supplement: Multimedia Appendix 1 [file jmir_v27i1e55492_app1.docx]

# **Complete Blood Count and MDW-based Machine Learning Algorithms for Sepsis Detection: a Multicentric Development and External Validation Study**

## **Supplementary Figures and Tables**


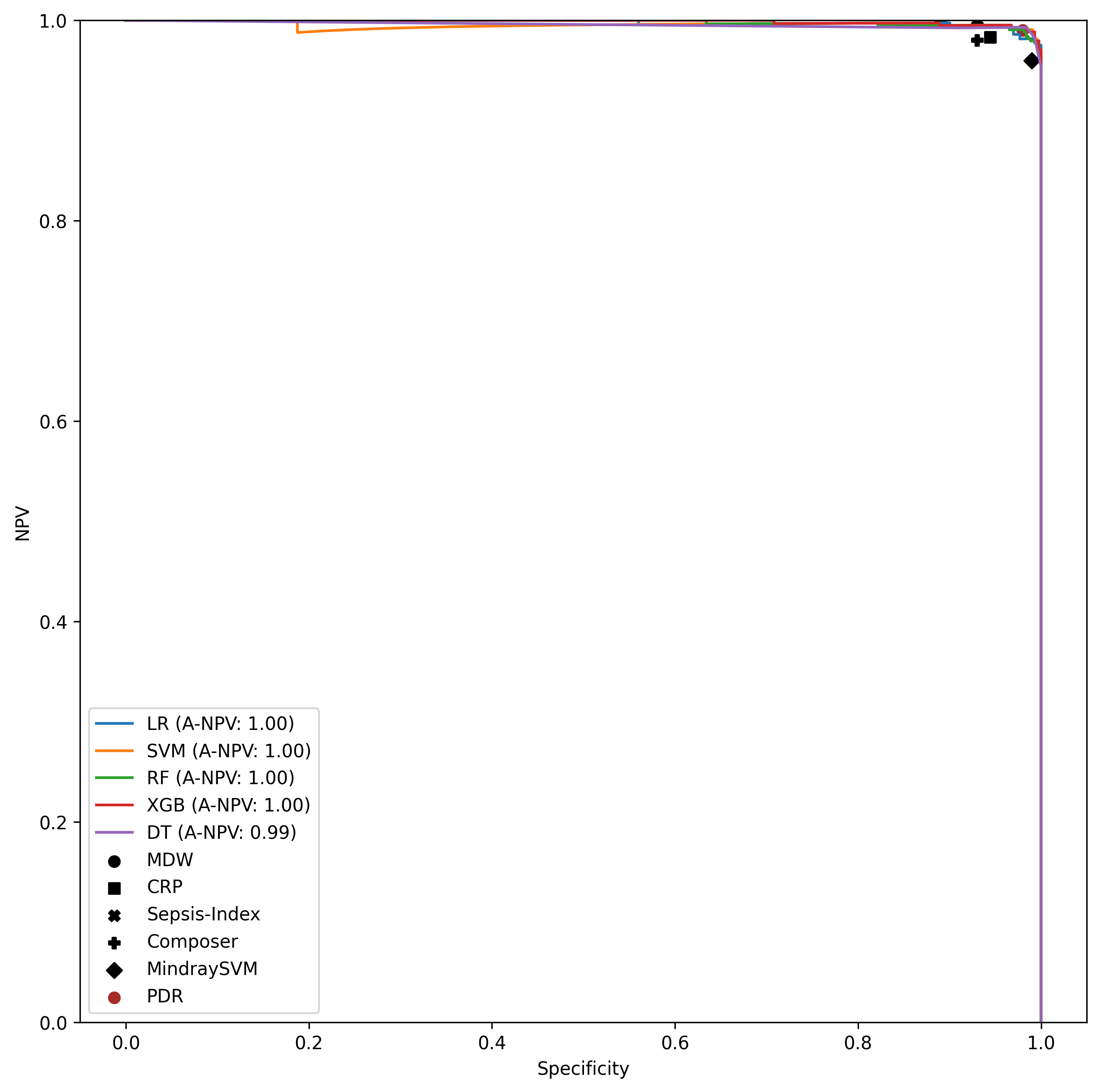


**Figure S1. Specificity-NPV curves for the models on the PA-ED internal validation dataset, along with their A-NPV value. In the plot we also report about the specificity and NPV of five baselines: binary thresholds based on the MDW and CRP parameters, a binary threshold based on the Sepsis-Index parameter, the COMPOSER model [25], and the SVM model developed in [32].**


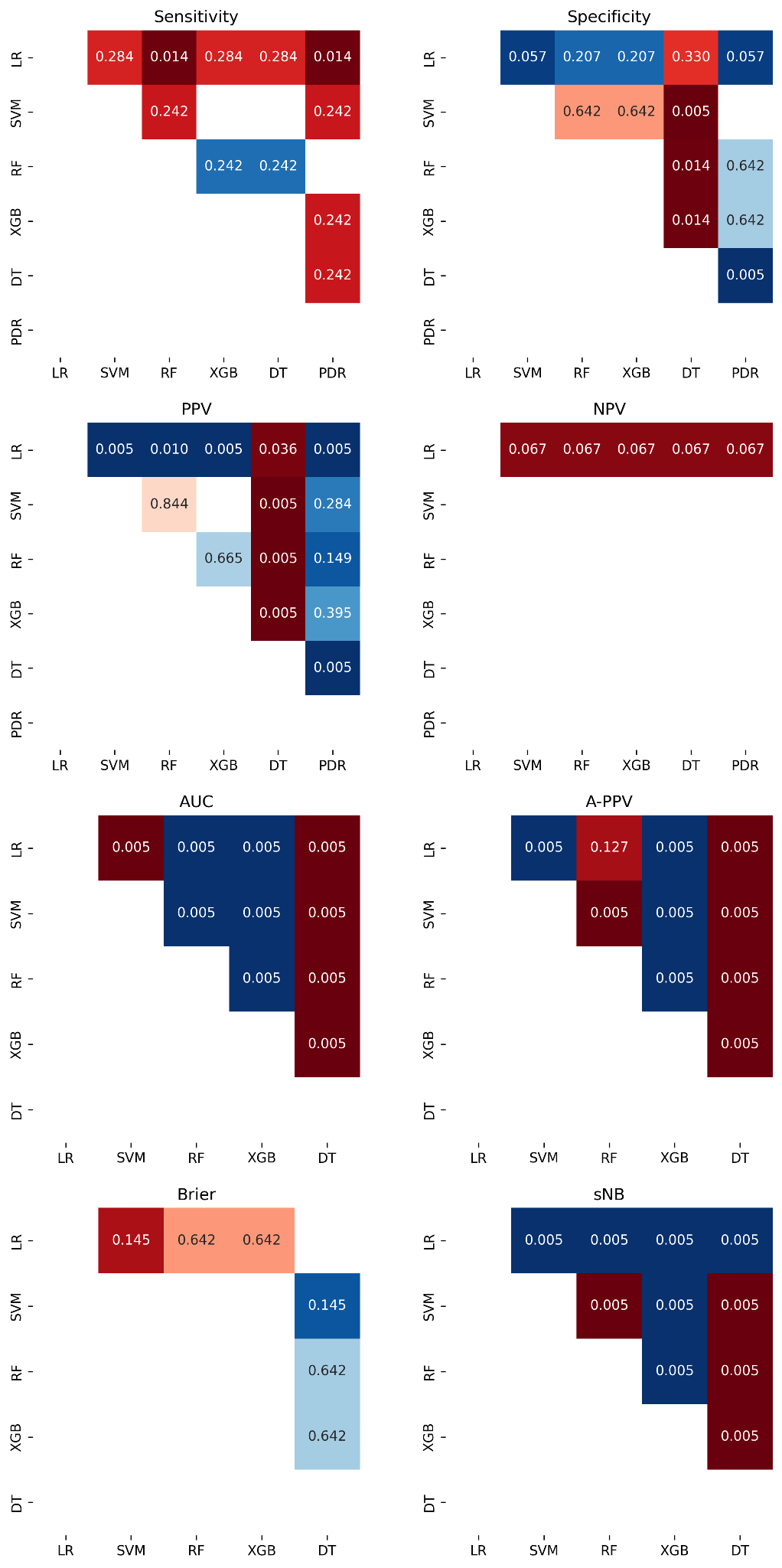
**Figure S2.** Heatmap of the adjusted p-values for the comparison among ML models on the internal validation. Red color denotes that the ML model on the corresponding row was better than the ML model on the corresponding column, vice versa blue color is used. In both cases, darker shades denote smaller p-values. P-values equal to 1 are not depicted. P-values were computed using a two-tailed chi-square test for difference in means.


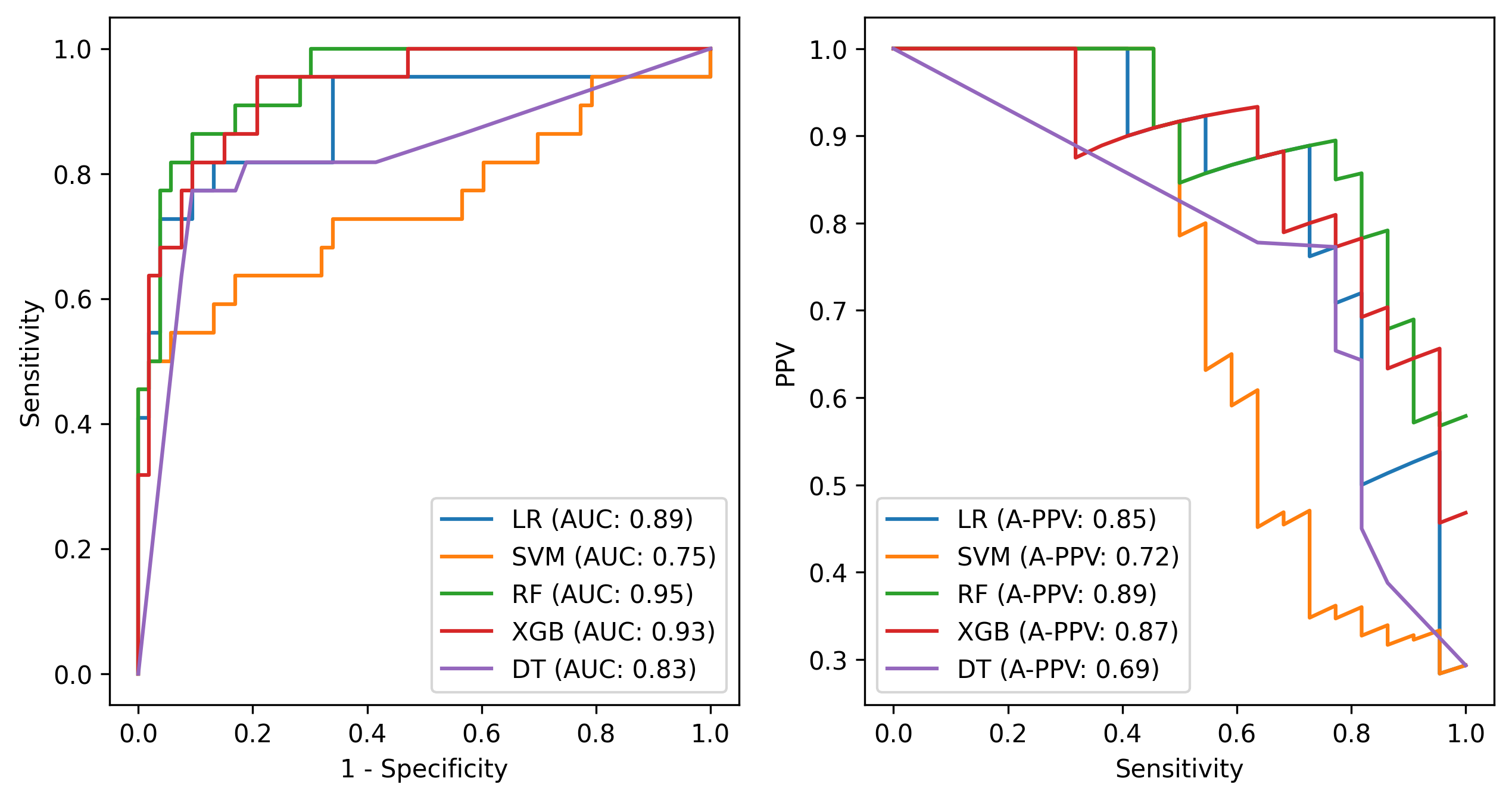
**Figure S3.** ROC (left) and Sensitivity-PPV (right) curves for the models, along with their AUC and A-PPV value, on the PA-ICU external validation dataset.


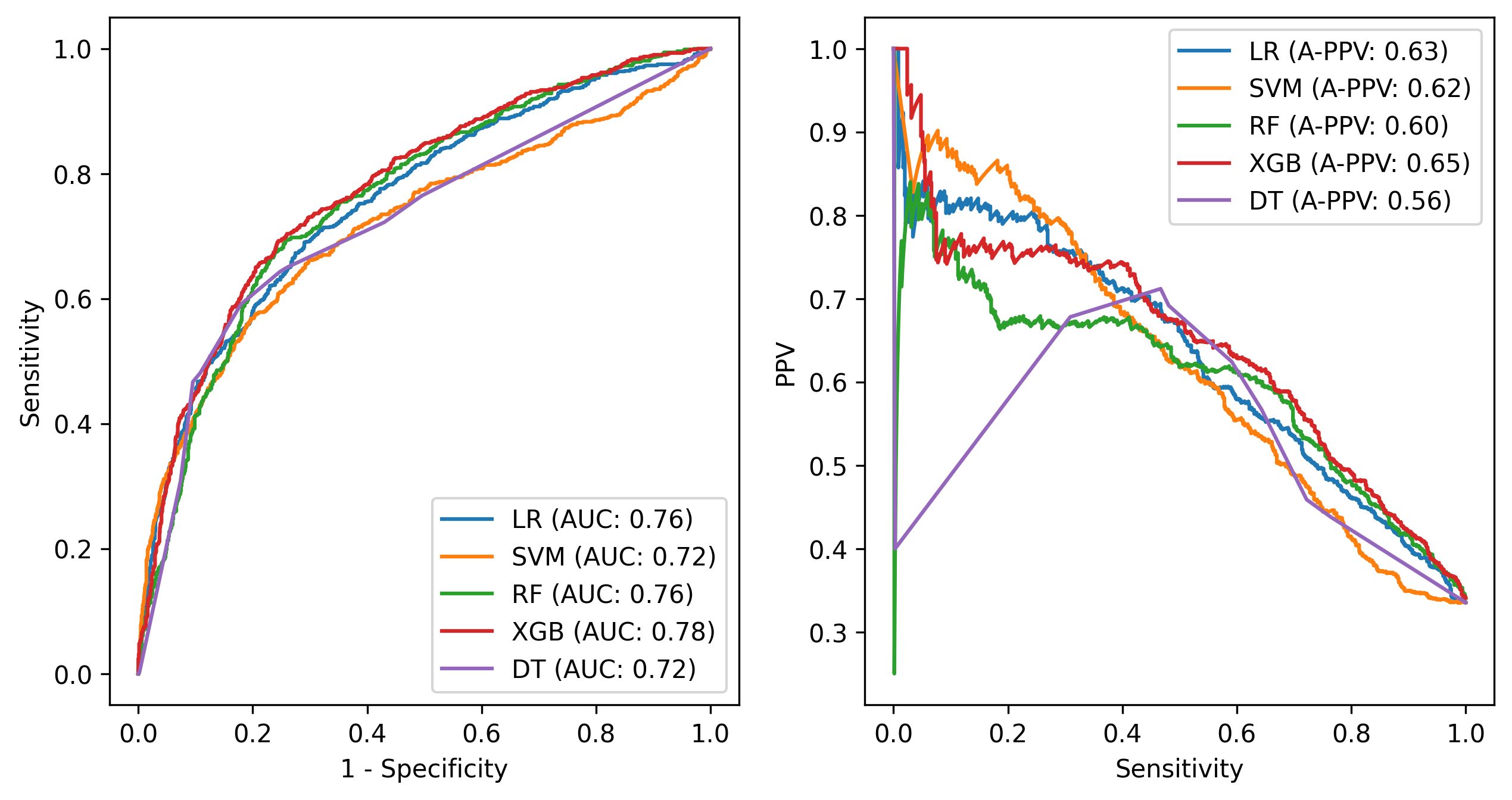
**Figure S4.** ROC (left) and Sensitivity-PPV (right) curves for the models, along with their AUC and A-PPV value, on the PD-ICU external validation dataset.


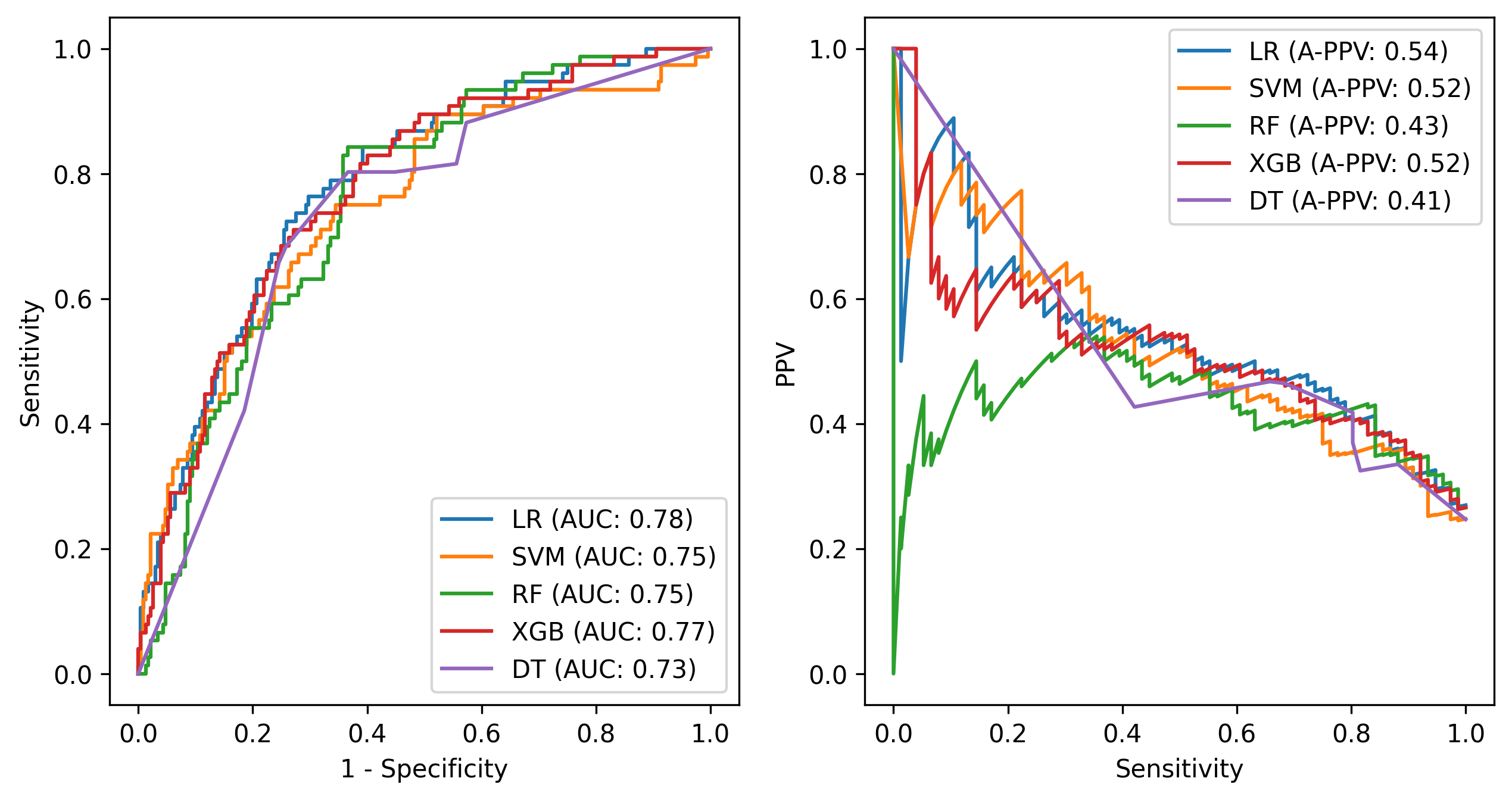
**Figure S5.** ROC (left) and Sensitivity-PPV (right) curves for the models, along with their AUC and A-PPV value, on the AR-ED external validation dataset.


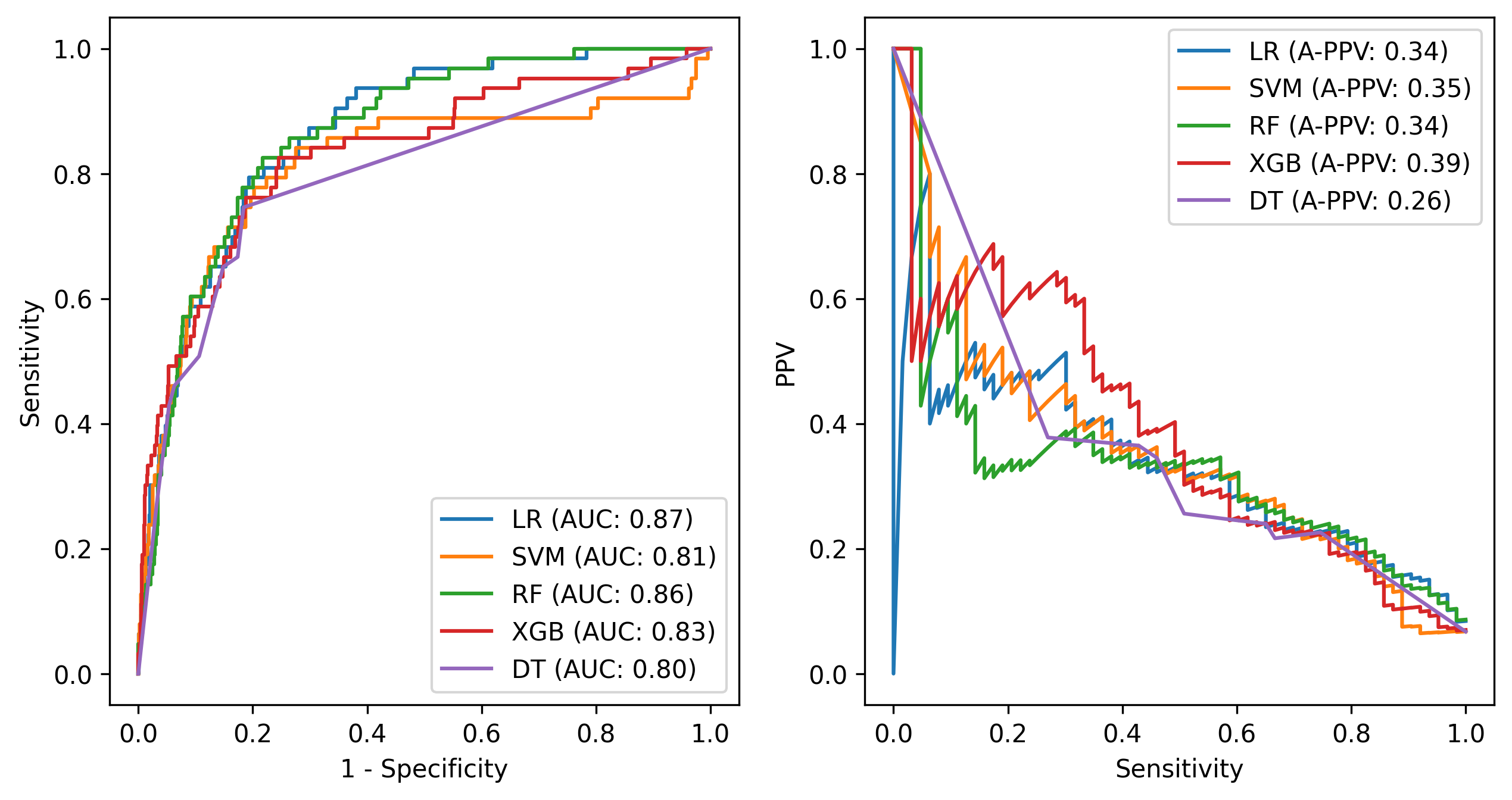
**Figure S6.** ROC (left) and Sensitivity-PPV (right) curves for the models, along with their AUC and A-PPV value, on the UD-ED external validation dataset.


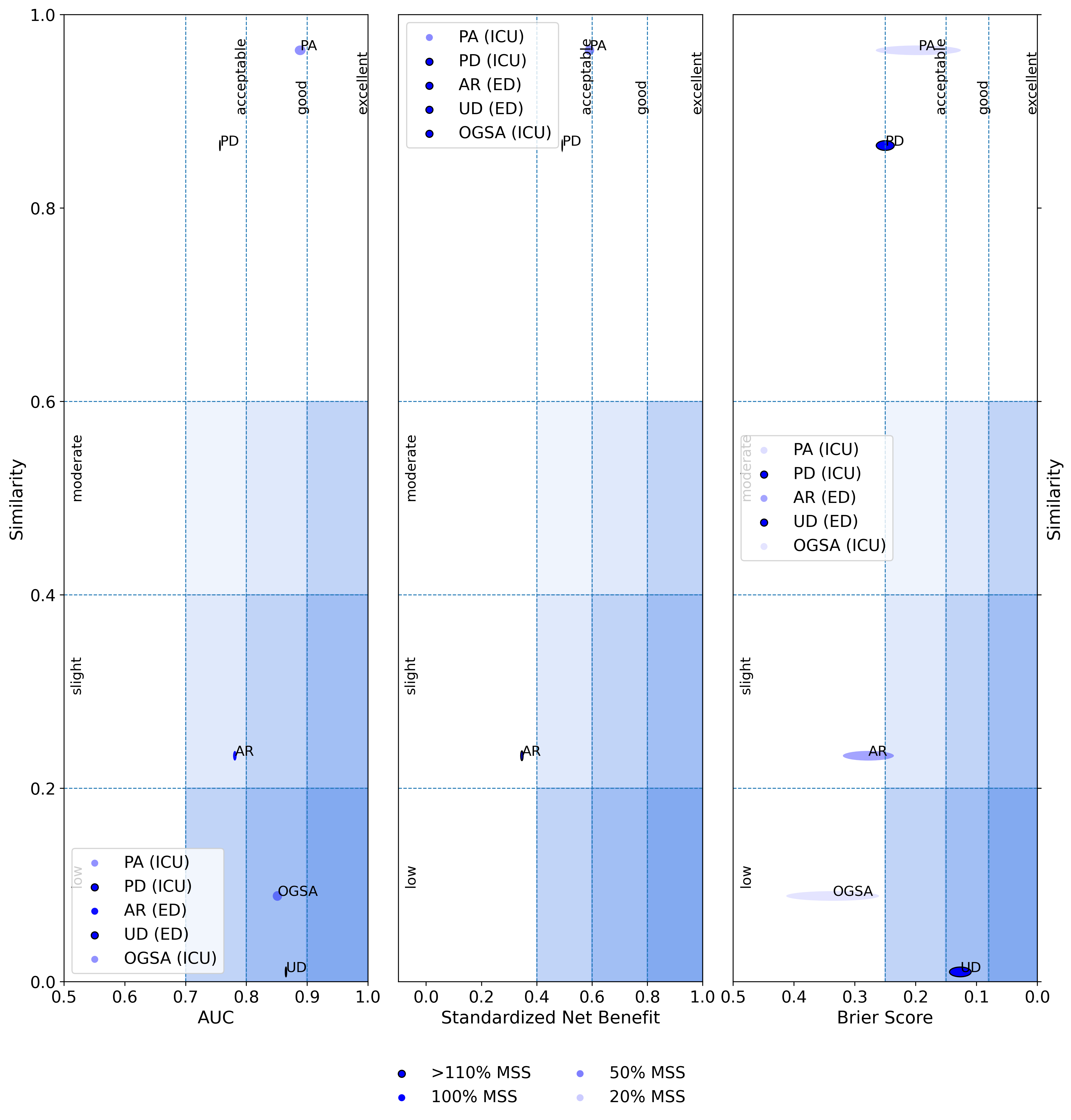


**Figure S7.** External performance diagram for the LR model on the external datasets. The diagram has been produced with the tool available at <https://mudilab.github.io/dss-quality-assessment/>.


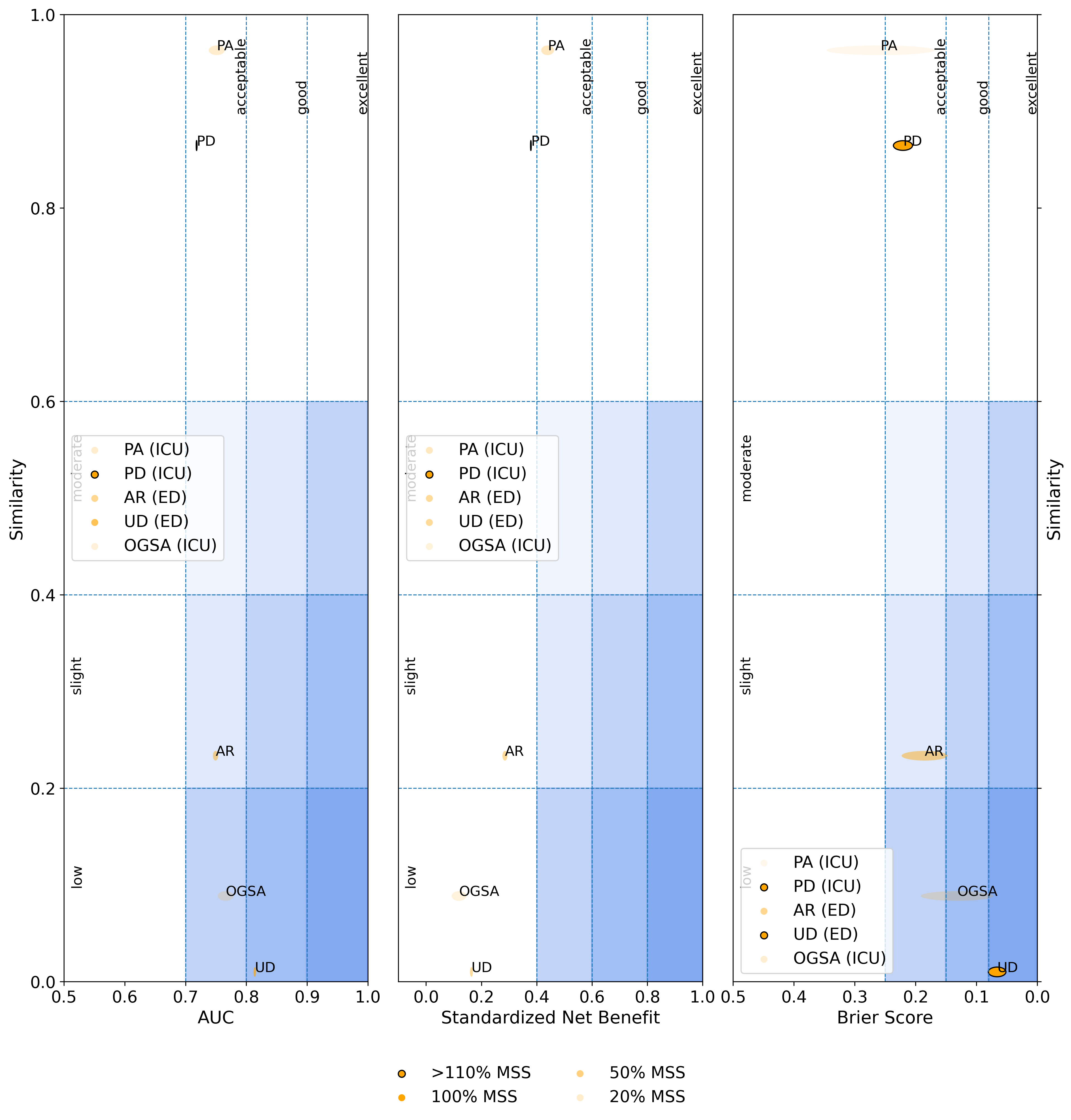


**Figure S8.** External performance diagram for the SVM model on the external datasets. The diagram has been produced with the tool available at <https://mudilab.github.io/dss-quality-assessment/>.


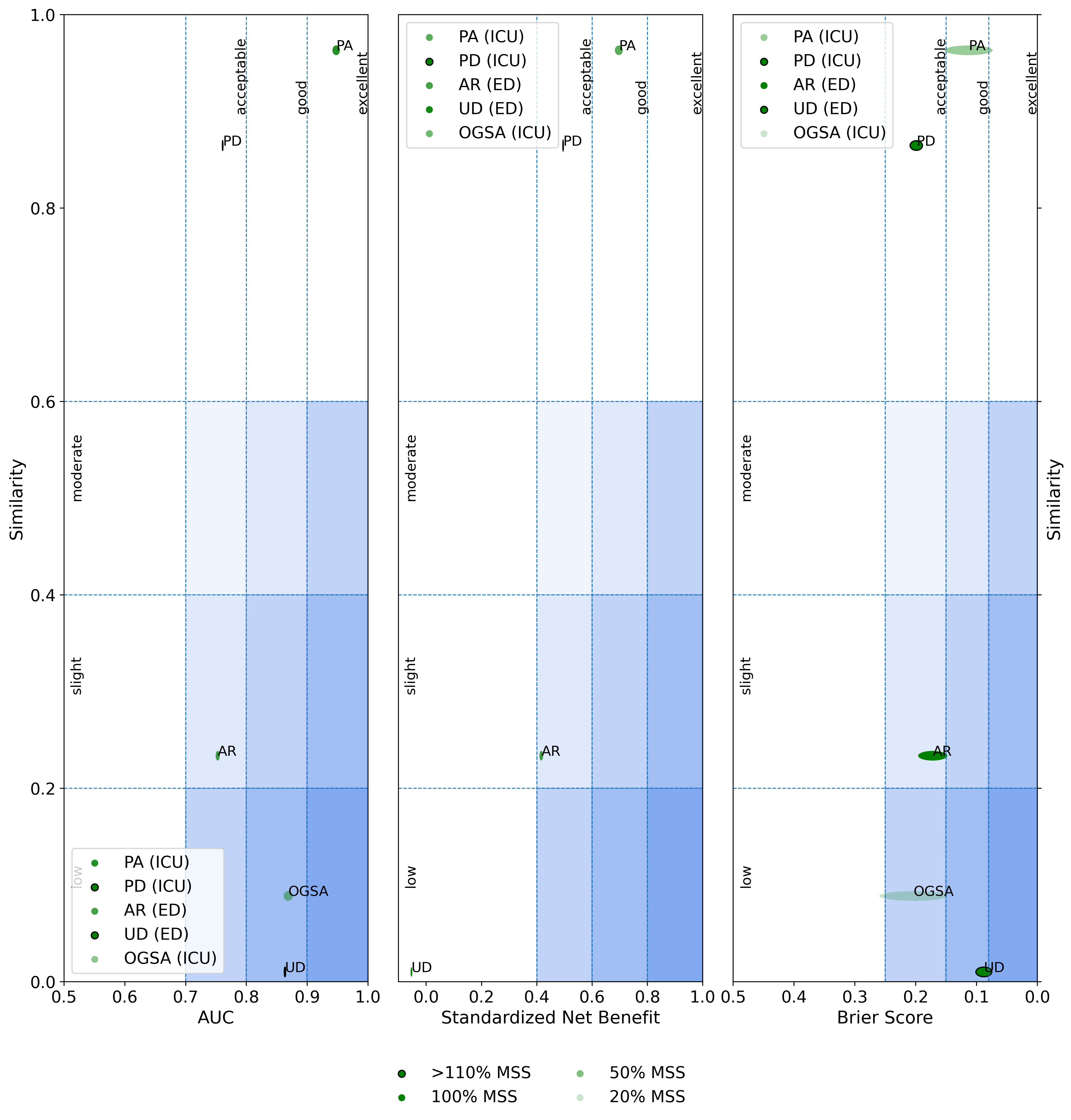


**Figure S9.** External performance diagram for the RF model on the external datasets. The diagram has been produced with the tool available at <https://mudilab.github.io/dss-quality-assessment/>.


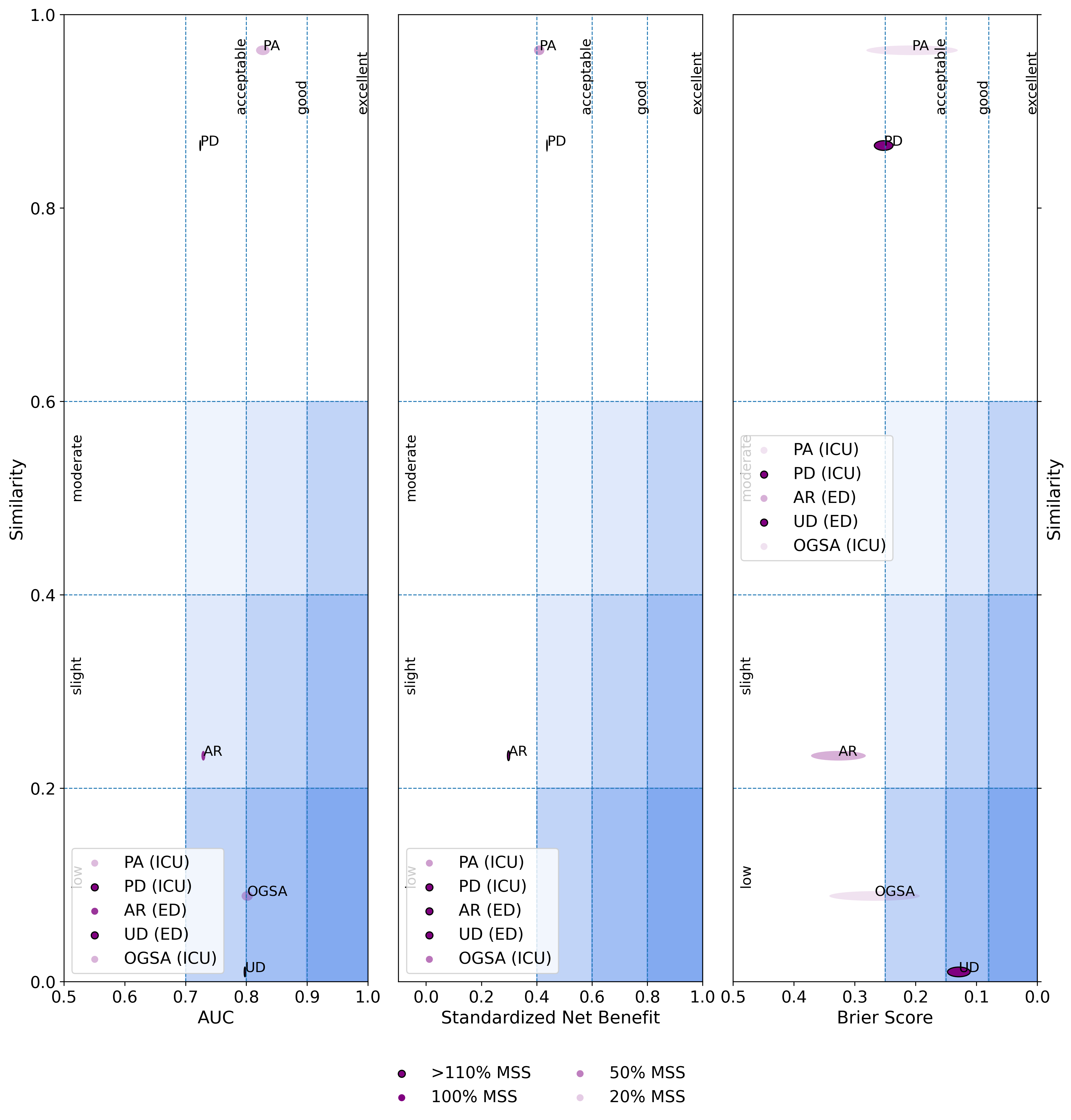


**Figure S10.** External performance diagram for the DT model on the external datasets. The diagram has been produced with the tool available at <https://mudilab.github.io/dss-quality-assessment/>.


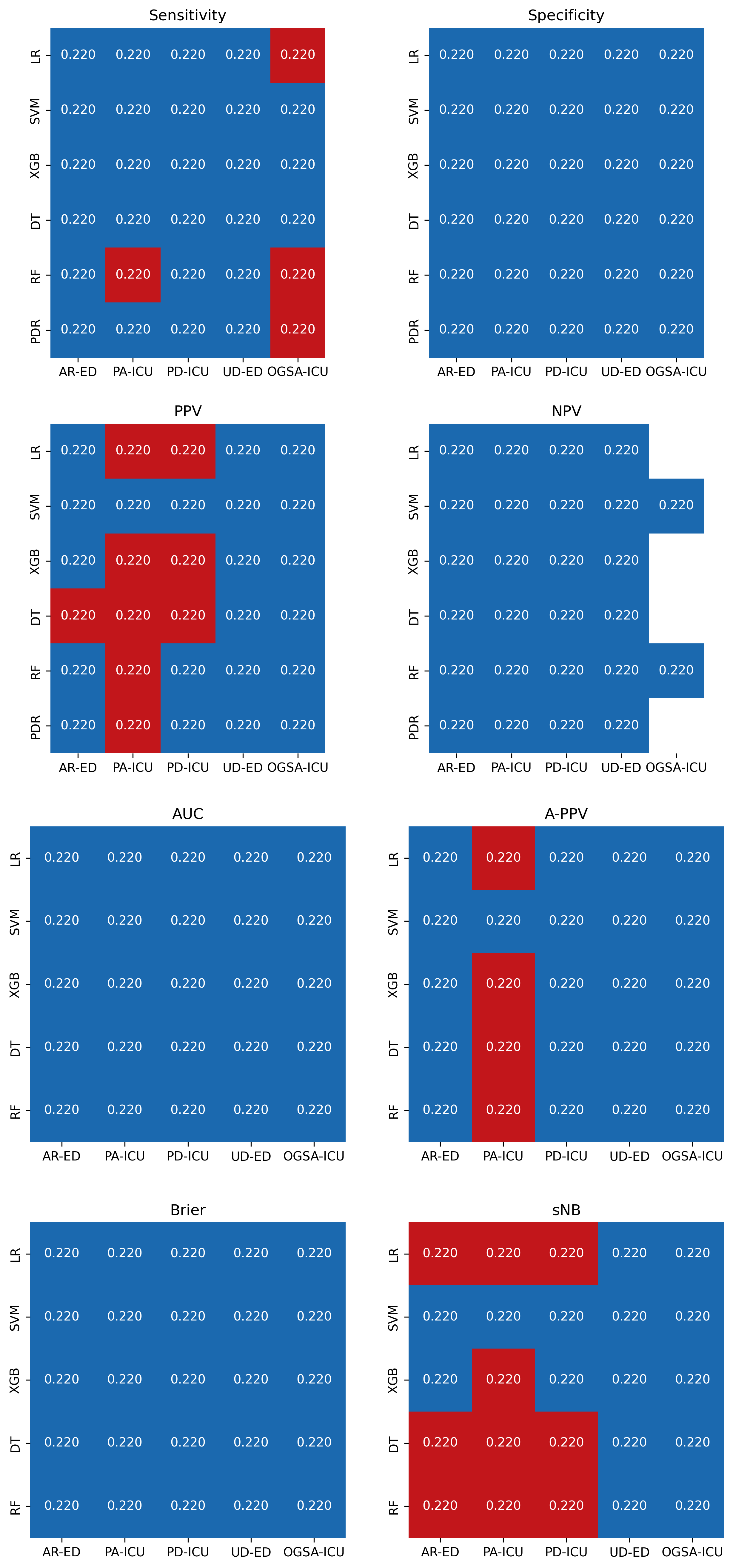


**Figure S11.** Heatmap of the adjusted p-values for the comparison between internal validation and external validation performance, for each ML model and external validation dataset. Red color denotes that the ML was better on the external dataset than on the internal one, vice versa blue color is used. In both cases, darker shades denote smaller p-values. P-values equal to 1 are not depicted. P-values were computed using a two-tailed chi-square test for difference in means.


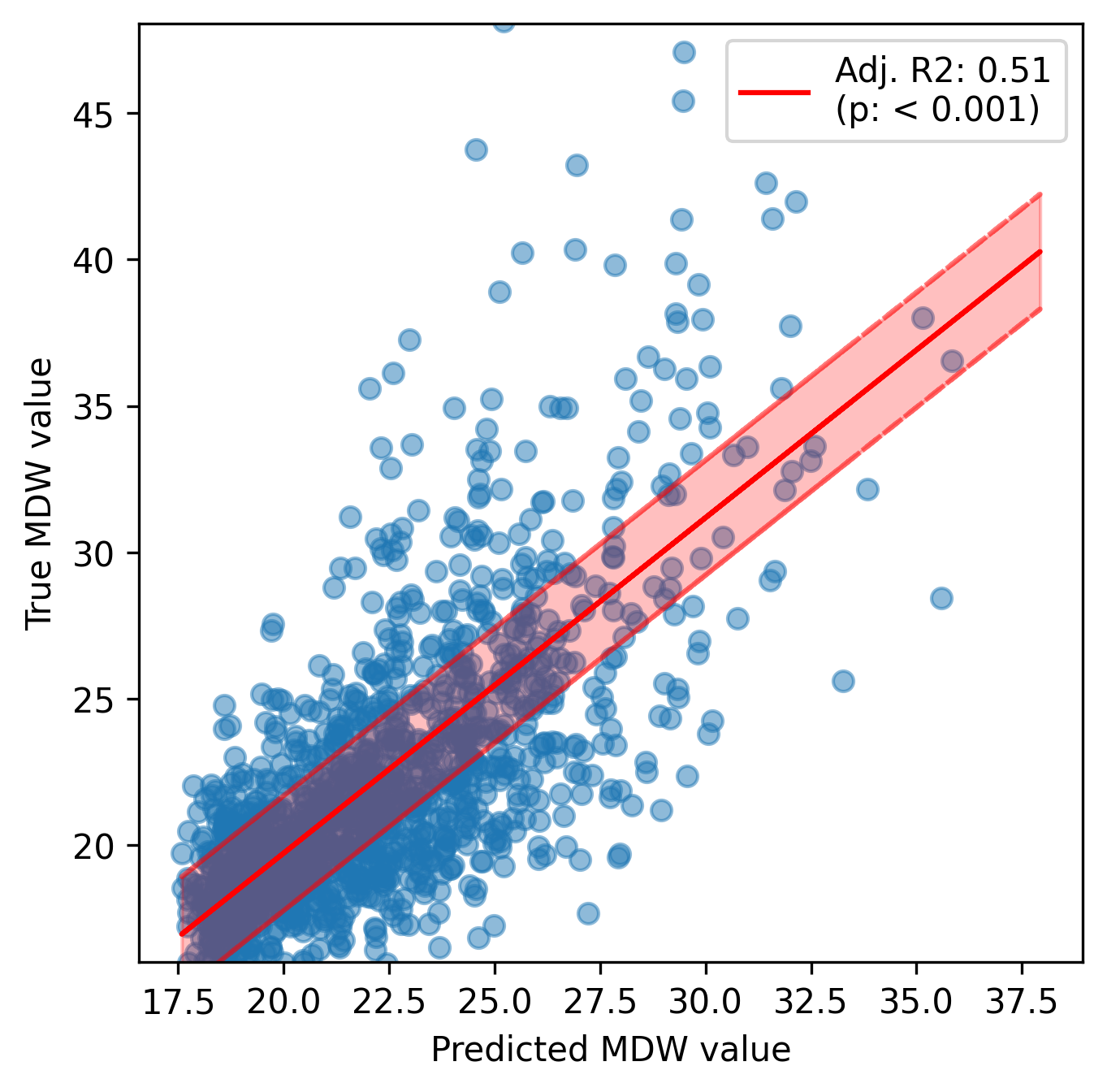
**Figure S12.** Regression analysis of the MDW variable imputation on the external validation cohorts. The red line represents the linear regression line for the correlation between the predicted and true MDW values, while the shaded red band represents the corresponding 95% prediction interval.


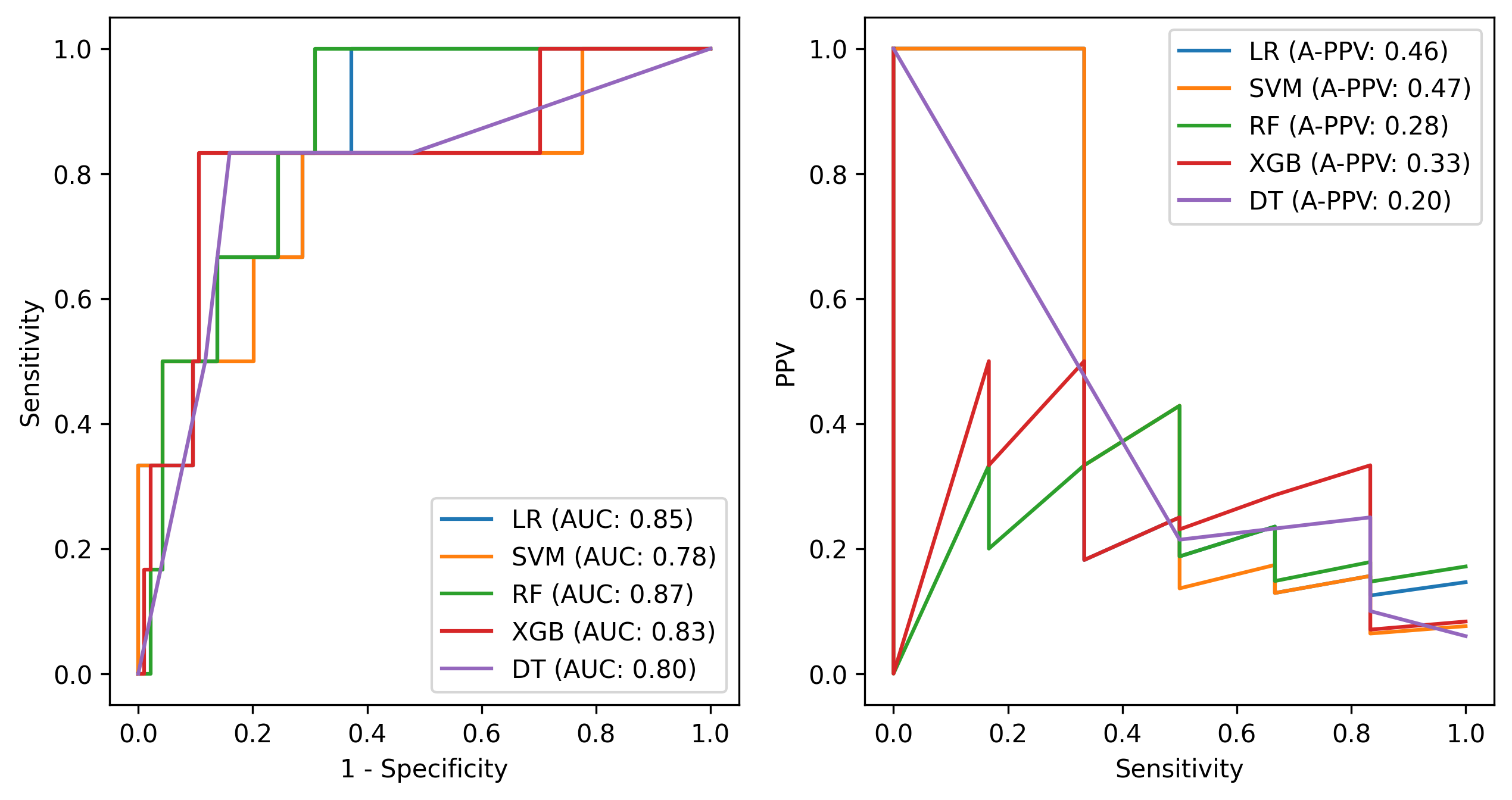
**Figure S13.** ROC (left) and Sensitivity-PPV (right) curves for the models, along with their AUC and A-PPV value, on the OGSA-ICU non-MDW validation dataset.


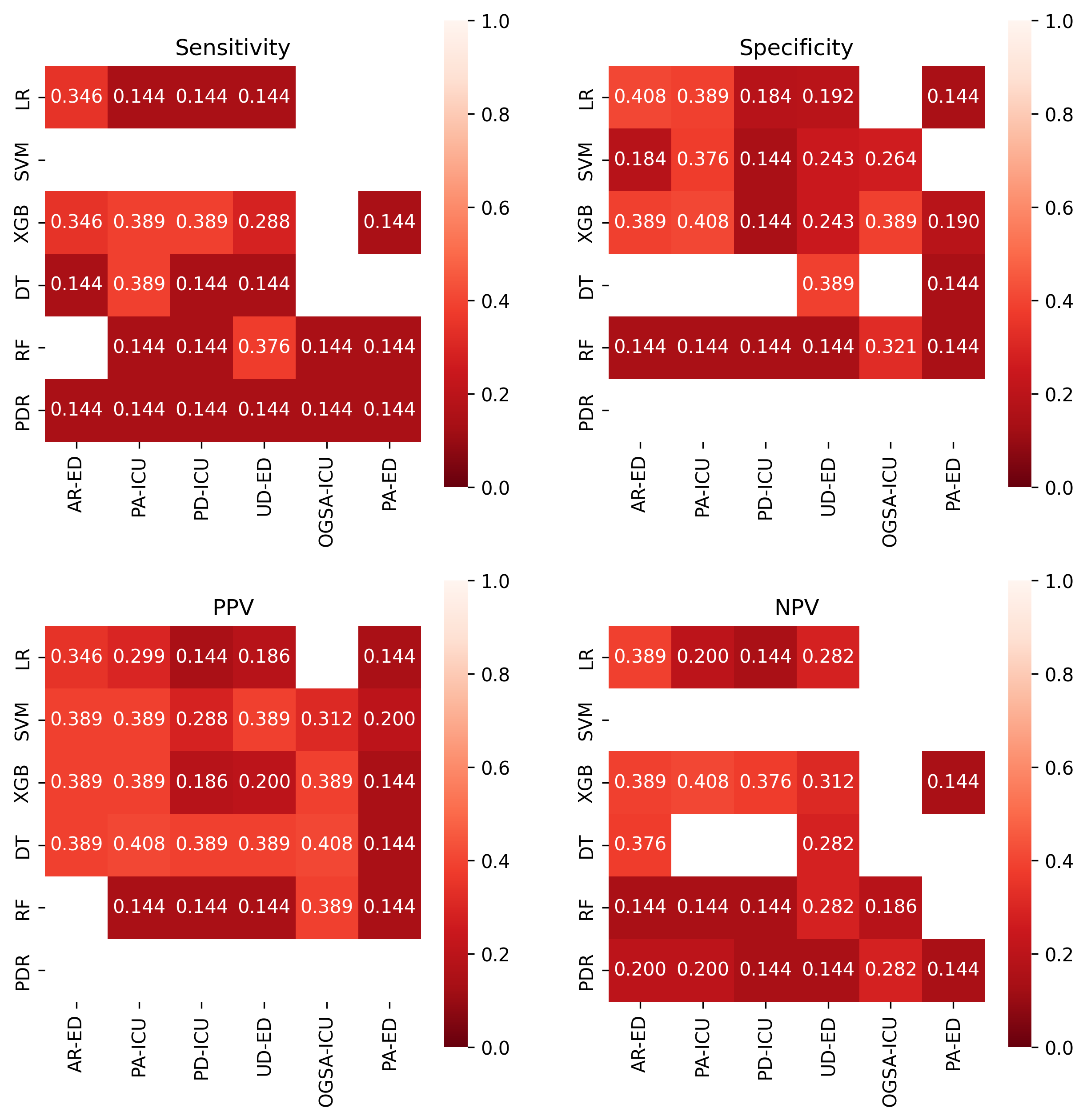


**Figure S14.** Heatmap of the adjusted p-values for the comparison among standard ML models and the corresponding cautious classifiers. Darker shades of red color denote smaller p-values for the one-tailed hypothesis that the cautious classifier was better than corresponding standard ML model. P-values were computed using a one-tailed chi-square test for difference in means.

**Table S1.** Hyper-parameters for the developed models.

| **Hyper-parameter** | **LR** | **SVM** | **RF** | **XGB** | **DT** |
| --- | --- | --- | --- | --- | --- |
|  |  |  |  |  |  |
| **penalty** | l2, l1, elasticnet |  |  |  |  |
| **max_iter** | 10000 | 1000 |  |  |  |
| **C** | Uniform(0.5,2) | Uniform(0,1) |  |  |  |
| **solver** | saga |  |  |  |  |
| **l1_ratio** | Uniform(0,1) |  |  |  |  |
| **random_state** | 0 | | | | |
| **n_features_to_select** | Uniform(5,19) | | | | |
| **class_weight** | balanced, None | | |  | balanced, None |
| **scaler** | min-max, standard, yeo-johnson, max-abs, normalize, robust, None | | | | None |
| **criterion** |  |  | gini, entropy |  | gini, entropy |
| **splitter** |  |  |  |  | best, random |
| **max_depth** |  |  | Uniform(1,100) | | Uniform(3,5) |
| **kernel** |  | linear, rbf, sigmoid, poly |  |  |  |
| **degree** |  | Uniform(2,10) |  |  |  |
| **gamma** |  | auto, scale |  | Uniform(0,100) |  |
| **probability** |  | True |  |  |  |
| **n_estimators** |  |  | Uniform(10,1000) | |  |
| **max_features** |  |  | sqrt, log2 |  |  |
| **eta** |  |  |  | Uniform(0.01,0.2) |  |
| **subsample** |  |  |  | Uniform(0.5,1) |  |
| **lambda** |  |  |  | Uniform(0,5) |  |
| **alpha** |  |  |  | Uniform(0,5) |  |
| **scale_pos_weight** |  |  |  | Uniform(0,100) |  |

**Table S2.** Results of the developed machine learning models on the external datasets and the non–monocyte distribution width validation dataset, together with the corresponding 95% CIs.

| Model and dataset | | Sensitivity, (95% CI) | Specificity, (95% CI) | PPV^a^, (95% CI) | NPV^b^, (95% CI) | AUC^c^, (95% CI) | Brier, (95% CI) | A-PPV^d^, (95% CI) | HC^e^ sensitivity, (95% CI) | HC specificity, (95% CI) | HC PPV, (95% CI) | HC NPV, (95% CI) | Coverage, (95% CI) | sNB^f^, (95% CI) |
| --- | --- | --- | --- | --- | --- | --- | --- | --- | --- | --- | --- | --- | --- | --- |
| **Logistic regression** | | | | | | | | | | | | | | |
|  | AR-ED^g^ | 0.82 (0.04) | 0.61 (0.05) | 0.41 (0.05) | 0.91 (0.03) | 0.78 (0) | 0.28 (0) | 0.54 (0) | 0.85 (0.04) | 0.62 (0.05) | 0.45 (0.06) | 0.92 (0.03) | 0.80 (0.04) | 0.35 (0) |
|  | OGSA-ICU^h^ | 1 (0) | 0.60 (0.10) | 0.14 (0.07) | 1 (0) | 0.83 (0.02) | 0.36 (0.03) | 0.23 (0.02) | 1 (0) | 0.50 (0.10) | 0.12 (0.06) | 1 (0) | 0.75 (0.08) | –2.28 |
|  | PA-ICU^i^ | 0.82 (0.09) | 0.66 (0.11) | 0.50 (0.11) | 0.90 (0.07) | 0.89 (0) | 0.20 (0.01) | 0.85 (0) | 0.95 (0.05) | 0.71 (0.10) | 0.60 (0.11) | 0.97 (0.04) | 0.80 (0.09) | 0.59 (0.01) |
|  | PD-ICU^j^ | 0.72 (0.02) | 0.65 (0.02) | 0.51 (0.02) | 0.82 (0.02) | 0.76 (0) | 0.25 (0) | 0.63 (0) | 0.77 (0.02) | 0.68 (0.02) | 0.57 (0.02) | 0.84 (0.02) | 0.77 (0.02) | 0.49 (0) |
|  | UD-ED^k^ | 0.65 (0.03) | 0.85 (0.02) | 0.24 (0.03) | 0.97 (0.01) | 0.87 (0) | 0.13 (0) | 0.34 (0) | 0.74 (0.03) | 0.88 (0.02) | 0.28 (0.03) | 0.98 (0.01) | 0.90 (0.02) | –0.13 |
| **Support vector machine** | | | | | | | | | | | | | | |
|  | AR-ED | 0.66 (0.05) | 0.73 (0.05) | 0.45 (0.06) | 0.87 (0.04) | 0.75 (0) | 0.19 (0) | 0.52 (0) | 0.61 (0.05) | 0.80 (0.04) | 0.48 (0.06) | 0.87 (0.04) | 0.91 (0.03) | 0.29 (0) |
|  | OGSA-ICU | 0.83 (0.07) | 0.70 (0.09) | 0.15 (0.07) | 0.99 (0.02) | 0.79 (0.02) | 0.14 (0.01) | 0.33 (0.03) | 0.83 (0.07) | 0.79 (0.08) | 0.22 (0.08) | 0.99 (0.02) | 0.90 (0.06) | –0.56 (0.09) |
|  | PA-ICU | 0.73 (0.10) | 0.55 (0.11) | 0.40 (0.11) | 0.83 (0.09) | 0.75 (0.01) | 0.26 (0.01) | 0.72 (0.01) | 0.70 (0.10) | 0.62 (0.11) | 0.44 (0.11) | 0.83 (0.09) | 0.89 (0.07) | 0.44 (0.01) |
|  | PD-ICU | 0.62 (0.02) | 0.74 (0.02) | 0.55 (0.02) | 0.80 (0.02) | 0.72 (0) | 0.22 (0) | 0.62 (0) | 0.57 (0.02) | 0.80 (0.02) | 0.57 (0.02) | 0.80 (0.02) | 0.91 (0.01) | 0.38 (0) |
|  | UD-ED | 0.59 (0.03) | 0.91 (0.02) | 0.31 (0.03) | 0.97 (0.01) | 0.81 (0) | 0.07 (0) | 0.35 (0) | 0.54 (0.03) | 0.93 (0.02) | 0.32 (0.03) | 0.97 (0.01) | 0.97 (0.01) | 0.16 (0) |
| **Random forest** | | | | | | | | | | | | | | |
|  | AR-ED | 0.55 (0.06) | 0.78 (0.05) | 0.45 (0.06) | 0.84 (0.04) | 0.75 (0) | 0.17 (0) | 0.43 (0) | 0.08 (0.03) | 0.97 (0.02) | 0.20 (0.04) | 0.92 (0.03) | 0.49 (0.06) | 0.42 (0) |
|  | OGSA-ICU | 0.83 (0.07) | 0.69 (0.09) | 0.15 (0.07) | 0.98 (0.02) | 0.86 (0.01) | 0.21 (0.02) | 0.28 (0.02) | 1 (0) | 0.75 (0.09) | 0.20 (0.08) | 1 (0) | 0.67 (0.09) | –1.61 |
|  | PA-ICU | 0.91 (0.07) | 0.79 (0.09) | 0.65 (0.11) | 0.95 (0.05) | 0.95 (0.00) | 0.11 (0) | 0.89 (0) | 1 (0) | 0.94 (0.05) | 0.89 (0.07) | 1 (0) | 0.69 (0.10) | 0.70 (0.01) |
|  | PD-ICU | 0.70 (0.02) | 0.73 (0.02) | 0.56 (0.02) | 0.83 (0.02) | 0.76 (0) | 0.20 (0) | 0.60 (0) | 0.76 (0.02) | 0.80 (0.02) | 0.67 (0.02) | 0.87 (0.01) | 0.55 (0.02) | 0.50 (0) |
|  | UD-ED | 0.60 (0.03) | 0.90 (0.02) | 0.29 (0.03) | 0.97 (0.01) | 0.86 (0) | 0.09 (0) | 0.34 (0) | 0.62 (0.03) | 0.94 (0.02) | 0.34 (0.03) | 0.98 (0.01) | 0.86 (0.02) | –0.05 |
| **Decision tree** | | | | | | | | | | | | | | |
|  | AR-ED | 0.80 (0.04) | 0.55 (0.06) | 0.37 (0.05) | 0.90 (0.03) | 0.73 (0) | 0.33 (0) | 0.41 (0) | 0.87 (0.04) | 0.52 (0.06) | 0.40 (0.05) | 0.92 (0.03) | 0.84 (0.04) | 0.30 (0) |
|  | OGSA-ICU | 0.83 (0.07) | 0.68 (0.09) | 0.14 (0.07) | 0.98 (0.02) | 0.79 (0.02) | 0.28 (0.02) | 0.18 (0.02) | 0.83 (0.07) | 0.62 (0.09) | 0.15 (0.07) | 0.98 (0.03) | 0.83 (0.07) | −1.72 |
|  | PA-ICU | 0.82 (0.09) | 0.79 (0.09) | 0.62 (0.11) | 0.91 (0.06) | 0.83 (0.01) | 0.21 (0.01) | 0.69 (0.01) | 0.86 (0.08) | 0.70 (0.10) | 0.64 (0.11) | 0.88 (0.07) | 0.72 (0.10) | 0.41 (0.01) |
|  | PD-ICU | 0.65 (0.02) | 0.74 (0.02) | 0.56 (0.02) | 0.81 (0.02) | 0.72 (0) | 0.25 (0) | 0.56 (0) | 0.73 (0.02) | 0.67 (0.02) | 0.57 (0.02) | 0.81 (0.02) | 0.79 (0.02) | 0.44 (0) |
|  | UD-ED | 0.65 (0.03) | 0.85 (0.02) | 0.24 (0.03) | 0.97 (0.01) | 0.80 (0) | 0.13 (0) | 0.26 (0) | 0.70 (0.03) | 0.86 (0.02) | 0.25 (0.03) | 0.98 (0.01) | 0.94 (0.02) | –0.11 |
| **eXtreme gradient boosting** | | | | | | | | | | | | | | |
|  | AR-ED | 0.61 (0.05) | 0.78 (0.05) | 0.48 (0.06) | 0.86 (0.04) | 0.77 (0) | 0.21 (0) | 0.52 (0) | 0.65 (0.05) | 0.80 (0.04) | 0.50 (0.06) | 0.88 (0.04) | 0.86 (0.04) | 0.40 (0) |
|  | OGSA-ICU | 0.83 (0.07) | 0.71 (0.09) | 0.16 (0.07) | 0.99 (0.02) | 0.82 (0.02) | 0.25 (0.02) | 0.32 (0.02) | 0.83 (0.07) | 0.73 (0.09) | 0.18 (0.08) | 0.98 (0.02) | 0.92 (0.05) | –0.89 |
|  | PA-ICU | 0.82 (0.09) | 0.85 (0.08) | 0.69 (0.10) | 0.92 (0.06) | 0.93 (0) | 0.13 (0) | 0.87 (0) | 0.86 (0.08) | 0.86 (0.08) | 0.72 (0.10) | 0.93 (0.06) | 0.95 (0.05) | 0.71 (0.01) |
|  | PD-ICU | 0.65 (0.02) | 0.79 (0.02) | 0.61 (0.02) | 0.82 (0.02) | 0.78 (0) | 0.22 (0) | 0.65 (0) | 0.66 (0.02) | 0.82 (0.02) | 0.64 (0.02) | 0.83 (0.02) | 0.88 (0.01) | 0.53 (0) |
|  | UD-ED | 0.52 (0.03) | 0.91 (0.02) | 0.30 (0.03) | 0.96 (0.01) | 0.83 (0) | 0.10 (0) | 0.39 (0) | 0.55 (0.03) | 0.93 (0.02) | 0.34 (0.03) | 0.97 (0.01) | 0.96 (0.01) | 0.09 (0) |
| **Partial Decision Rule (PDR)** | | | | | | | | | | | | | | |
|  | AR-ED | 0.79 (0.05) | 0.64 (0.05) | 0.42 (0.06) | 0.90 (0.03) | N/A | N/A | N/A | 0.90 (0.03) | 0.56 (0.06) | 0.42 (0.06) | 0.94 (0.03) | 0.84 (0.04) | N/A |
|  | OGSA-ICU | 0.83 (0.07) | 0.82 (0.07) | 0.23 (0.05) | 0.99 (0.02) | N/A | N/A | N/A | 1.00 (0.01) | 0.69 (0.09) | 0.25 (0.07) | 1.00 (0.01) | 0.60 (0.10) | N/A |
|  | PA-ICU | 0.77 (0.09) | 0.87 (0.08) | 0.71 (0.10) | 0.90 (0.07) | N/A | N/A | N/A | 0.94 (0.05) | 0.82 (0.09) | 0.71 (0.10) | 0.97 (0.04) | 0.77 (0.09) | N/A |
|  | PD-ICU | 0.58 (0.02) | 0.84 (0.02) | 0.64 (0.02) | 0.80 (0.02) | N/A | N/A | N/A | 0.78 (0.02) | 0.77 (0.02) | 0.64 (0.02) | 0.87 (0.01) | 0.71 (0.02) | N/A |
|  | UD-ED | 0.48 (0.03) | 0.90 (0.02) | 0.26 (0.03) | 0.96 (0.01) | N/A | N/A | N/A | 0.65 (0.03) | 0.89 (0.02) | 0.26 (0.03) | 0.98 (0.01) | 0.90 (0.02) | N/A |
